# Pedal acceleration time as a diagnostic and prognostic tool in peripheral artery disease: systematic review and meta-analysis

**DOI:** 10.1186/s12880-026-02493-y

**Published:** 2026-07-09

**Authors:** Mariana Rosado-Fonseca, Eduardo Silva, Tiago Ribeiro, Antónia Rocha-Melo, Daniela Santos-Silva, Mário Marques-Vieira, Piotr Myrcha, Hugo Ribeiro, João Rocha-Neves

**Affiliations:** 1 Faculty of Medicine, University of Porto, Alameda Prof. Hernâni Monteiro, Porto, 4200-319 Portugal; 2 Department of Angiology and Vascular Surgery, Unidade Local de Saúde de Coimbra, Coimbra, Portugal; 3 Department of Vascular Surgery, Unidade Local de Saúde – São José, Lisboa, Portugal; 4 Department of Palliative Care and Pain Medicine, Faculty of Medicine, University of Coimbra, Coimbra, Portugal; 5 Department of Angiology and Vascular Surgery, Unidade Local de Saúde de Braga, Braga, Portugal; 6 Department of General and Vascular Surgery, Faculty of Medicine, Medical University of Warsaw, Warsaw, 02-091 Poland; 7 Department of General, Vascular and Oncological Surgery, Brodnowski Hospital, Warsaw, 03-242 Poland; 8 Community Palliative Care Support Team Gaia, Local Health Unit Gaia and Espinho, Vila Nova de Gaia, Portugal; 9 Department of Medicina da Comunidade, Informação e Decisão em Saúde, Faculty of Medicine, University of Porto, Porto, Portugal; 10 Centre for Innovative Biomedicine and Biotechnology, Coimbra, Portugal; 11 Department of Biomedicine, Unit of Anatomy, Faculty of Medicine, University of Porto, Porto, Portugal; 12 Department de Biomedicine, RISE-Health, Faculty of Medicine, University of Porto, Alameda Prof. Hernâni Monteiro, Porto, 4200-319 Portugal; 13 Department of Vascular Surgery, Unidade Local de Saúde – Alto Ave, Guimarães, Portugal

**Keywords:** Wound management, Foot ulcer, Ultrasonography doppler, Limb salvage, Diabetic foot, Ankle-brachial index, Chronic limb-threatening ischaemia

## Abstract

**Background:**

Wound healing in chronic limb-threatening ischaemia in peripheral artery disease (PAD) remains clinically challenging. Accurate perfusion assessment may predict limb outcomes. Standard measures ankle-brachial index (ABI) and transcutaneous oxygen pressure have limitations. The ABI, the guideline-recommended first-line diagnostic tool, has well-recognised limitations in patients. Pedal acceleration time (PAT), measured by duplex ultrasound from onset to peak arterial systole in the pedal arteries, shows promise as a prognostic marker, though its diagnostic and prognostic performance has not been fully validated. This study aimed to perform a systematic review and meta-analysis evaluating PAT’s diagnostic accuracy compared with ABI in detecting lower-limb ischaemia. Secondary objectives included assessing PAT’s prognostic value for wound healing and amputation risk.

**Methods:**

Three electronic databases MEDLINE, Scopus, and Web of Science, were systematically searched for studies evaluating PAT values and wound healing in PAD patients. Wound healing incidence and PAT-ABI correlations were pooled according to Preferred Reporting Items for Systematic reviews and Meta-Analyses of Diagnostic Test Accuracy studies (PRISMA-DTA) guidelines. Pooled diagnostic indices, including sensitivity, specificity, positive and negative likelihood ratios, and diagnostic odds ratio with 95% confidence intervals were calculated. Study quality was assessed using the National Heart, Lung, and Blood Institute (NHLBI) Study Quality Assessment Tool for observational cohort and cross-sectional designs.

**Results:**

Seventeen studies (1749 patients) were included as following: 8 cross-sectional, 1 observational prospective, 3 observational retrospective, 4 cohort (3 retrospective, 1 prospective), and 1 case-control study. PAT demonstrated good diagnostic accuracy with pooled 0.72 (95% CI 0.68–0.76) sensitivity, 0.84 (95% CI 0.79–0.88) specificity, 4.38 (95% CI 3.35–5.73) positive likelihood ratio, 0.25 (95% CI 0.19–0.33) negative likelihood ratio, and 14.85 (95% CI 10.02–22.01) diagnostic odds ratio, indicating robust discrimination compared to ABI. The selected studies, referring to the period 2019–2025, reported an association between higher PAT values and worse wound healing outcomes and increased amputation risk. Due to insufficient and non-standardised data, PAT’s prognostic pooled analysis for wound healing was not feasible.

**Conclusion:**

PAT accurately detected clinically significant lower-limb ischaemia compared to ABI. Faster PAT values correlated to higher ABI and favorable wound healing outcomes, whereas prolonged PAT indicated higher risk of non-healing wounds and major amputation. These results support PAT as a useful duplex-based complement to existing perfusion tests.

**Supplementary Information:**

The online version contains supplementary material available at https://doi.org/10.1186/s12880-026-02493-y.

## Introduction

Peripheral artery disease (PAD) is a widespread, progressive atherosclerotic condition affecting over 200 million people worldwide [1]. It is characterised by the narrowing or occlusion of peripheral arteries leading to significant impairment of blood flow to the lower extremities [1, 2]. PAD prevalence is predicted to increase substantially, with the possibility of reaching approximately 360 million individuals over the next 25 years [3]. This vascular disorder frequently remains underdiagnosed, thereby contributing to a diminished quality of life and increased cardiovascular and limb-related mortality, and thus, represents a significant global health burden [3, 4]. The disease progresses insidiously, often going undetected until advanced stages, when functional limitation, tissue loss, or limb-threatening complications become apparent [2, 5].

The ankle-brachial index (ABI) is a well-known, non-invasive haemodynamic ratio, and is recommended as the first-line diagnostic test for PAD in the majority of societal guidelines [4, 6]. The Toe-Brachial Index is frequently used as a complementary perfusion assessment tool, particularly in patients with non-compressible vessels [7]. However, ABI measurements might be unreliable in the presence of medial artery calcification, leading to falsely elevated (> 1.40) ABI and diagnostic uncertainty, particularly in patients with diabetes mellitus, chronic kidney disease, or advanced arterial stiffness [1, 6, 8]. This limitation encourages clinicians to introduce complementary perfusion metrics into the evaluation of patients with lower-limb ischaemia.

PAT is a Doppler ultrasound-derived parameter that measures, in milliseconds, the interval from the onset of systolic flow to the systolic peak in distal pedal arteries, thereby quantifying the haemodynamic delay caused by proximal arterial stenosis or occlusion [9, 10]. In healthy limbs, PAT values are short, whereas progressive elongation is associated with increasing ischaemia severity, arterial stiffness, and impaired wound healing potential [11–13]. Previous studies have shown that PAT may allow grading of limb ischaemia severity and may be associated with clinically relevant outcomes, such as wound healing and limb salvage, even in cases of medial calcification or non-compressive vessels [9, 14]. Nevertheless, existing evidence is largely derived from small, retrospective, single-centre studies and has not been comprehensively evaluated.

Therefore, this systematic review and meta-analysis of patients with lower-limb ischaemia aims to evaluate PAT for the assessment of ischaemia in comparison with established perfusion tools such as ABI, thereby assessing diagnostic and prognostic performance. Secondary outcomes include assessing the prognostic value of PAT in predicting wound healing outcomes, evaluating its prognostic role in stratifying amputation risk, and determining its utility as a monitoring tool for revascularisation adequacy. Through these objectives, the present study seeks to clarify the potential role of PAT as a complementary tool for assessing lower-limb perfusion.

## Methods

This systematic review was conducted in accordance with the Preferred Reporting Items for a Systematic Review and Meta-analysis – Diagnostic Test Accuracy (PRISMA-DTA) Statement [15] and the A Measurement Tool to Assess Systematic Reviews version 2 (AMSTAR-2) critical appraisal tool [16]. The completed PRISMA-DTA checklist is provided in the supplementary material (Additional file 1, Supplemental Table 1). Due to the nature of this study, ethical approval from an institutional review board is not required. The review protocol has been registered at Prospero (reference: CRD420251035946).

### Selection criteria

Inclusion criteria comprised original human studies (except for systematic reviews and case series under 10 patients), evaluating PAT in the context of PAD. Eligible studies assessed PAT either as a diagnostic tool for lower-limb ischaemia or as a prognostic marker for limb-related outcomes, irrespective of whether PAT was measured pre-operatively, post-operatively, or at a single time point.

Studies reporting corresponding ABI values were included to allow correlation with PAT-derived perfusion metrics. Studies providing both pre- and post-operative PAT measurements were identified to explore associations between changes in PAT and clinical outcomes, including wound healing, limb salvage, major amputation, and revascularisation response. No restrictions were applied regarding publication date or language.

Clinically significant lower-limb ischemia was defined according to the reference standard applied in each primary study. For the diagnostic meta-analysis, ABI-defined ischemia, using study-specific thresholds consistent with guideline-recommended cut-offs, was treated as the reference standard, recognising that ABI is an imperfect surrogate, particularly in patients with non-compressible vessels.

### Search strategy

A systematic search was performed in three databases: Medline via PubMed search, Scopus, and Web of Science, in August 2025. The full search strategy, including search queries and keywords, is provided in Additional File 1 (Supplemental Table 2).

Additionally, the reference lists of the included primary studies and relevant available systematic reviews were screened (snowballing) for any further articles of possible interest.

### Study selection and data extraction

After duplicates removal, two authors (MRF and ES) have independently participated in the study selection. Disagreements on the choices were solved by the intervention of a third author (JRN). First, studies were selected by title and abstract, followed by full-text assessment.

Studies were excluded if they involved patients without PAD or patients exclusively suffering from acute limb ischaemia, or if PAT was not assessed in relation to ABI, wound healing, or major amputation. Case reports, editorials, narrative reviews, grey literature, and conference abstracts were excluded. When full texts were not publicly available, authors were contacted. MRF and ES also performed a full-text assessment. The selected studies were carefully revised to avoid repeated populations. In the case of duplicate populations, the most detailed analysis was included.

Data extraction was independently conducted by two authors (MRF and ES) using a purpose-built form. Extracted study characteristics included year of publication, country, recruitment centre, study design, recruitment period, number of participants, and limbs undergoing PAT measures, and follow-up duration. Participant demographics included age, gender distribution, and prevalence of cardiovascular comorbidities. These data are available in Tables 1 and 2.

**Table 1:** Characteristics and methodological quality of included studies

Authors	Journal	Pub Year	Design	Study Centre, Continent	Follow-up	Recruitment Period	GRADE Evaluation
Arévalo-Zamora et al.	Revista Angiología	2022	Prospective, Cross-Sectional	2 centres in Medellín, Colombia, America	No	1 year	⊕⊕○○Low
de Castro-Santos et al.	Vascular Medicine (London, England)	2023	Retrospective Cross-Sectional	University Hospital Risoleta Tolentino Neves, Brazil, America	No	06/2019 and 03/2020	⊕⊕○○Low
Del Valle et al.	Journal Of Vascular Ultrassound	2022	Prospective Cross-Sectional	Bronx Lebanon Hospital Center, NY, America	No	No	⊕⊕○○Low
Hart et al.	European Journal of Vascular and Endovascular Surgery	2024	Prospective Cohort	Waikato Hospital, Hamilton, New Zeeland, Oceania	1 year	(2,5 Years) 01/01/2020-30/6/2022	⊕⊕○○Low
Junaidi et al.	Chirurgia (Bucur)	2020	Retrospective Case-Control	Dr. Cipto Mangunkusumo General Hospital, Fatma Wati General Hospital, and Tangerang District General Hospital, Indonesia, Ásia	No	Not specified	⊕⊕○○Low
Mendes-Pinto et al.	Sociedade Brasileira De Angiologia E Cirurgia Vascular	2025	Retrospective Cross-Sectional	Hospital Felício Rocho, Belo Horizonte, Brazil, America	No	(4 months) 08/22 − 12/22	⊕⊕○○Low
Ochoa-Ayón et al.	Mexican Journal of Angiology	2022	Prospective Cohort	Regional Hospital of Morelia, Michoacán, México, America	07/2020-03/2022	8 months	⊕⊕○○Low
Pinelo et al.	Annals Of Vascular Surgery	2024	Prospective Cross-Sectional	Department of Angiology and Vascular Surgery, Santo António’s Hospital, Porto, Portugal, Europe	No	(1 month) 11/2022-12/2022	⊕⊕○○Low
Silva et al.	Portuguese Journal of Cardiac Thoracic and Vascular Surgery	2025	Retrospective Cross-Sectional	Department of Angiology and Vascular Surgery, Coimbra Local Health Unit, Coimbra, Portugal, Europe	No	(1year) throughout 2023	⊕⊕○○Low
Sommerset et al.	European Society of Vascular Surgery	2025	Prospective Cross-Sectional	PeaceHealth Vascular Surgery, Washington, and Baylor College of Medicine, Houston, USA; Sunnybrook Health Sciences Centre, Ontario, Canada, and Vascular Health Care, New South Wales, Australia, Oceania	No	06/2021-11/2022	⊕⊕○○Low
Spiliopoulos et al.	Journal Of Endovascular Therapy	2023	Prospective Observational	Attikon University General Hospital, Athens, Greece, Europe	1 year	(5 months) 10/2021-03/2022	⊕⊕○○Low
Teso et al.	Annals Of Vascular Surgery	2021	Retrospective Observational	PeaceHealth Southwest Medical Centre, Vancouver, Washington, EUA, America	12,8 ± 3,6 months	(2 years) 01/01/2018-31/12/2019	⊕⊕○○Low
Trihan et al.	Vascular Medicine (London, England)	2024	Retrospective Observational	Vascular Medicine Unit, Hospital of Cholet, Pays de la Loire, France, Europe	No	(4 months) 01/03/2022-01/07/2022	⊕⊕○○Low
Trihan et al.	Frontiers In Cardiovascular Medicine	2021	Retrospective Observational	Vascular Medicine Unit, University Hospital of Poitiers, Poitiers, France, Europe	1 year	Not specified	⊕⊕○○Low
Tun et al.	Annals Of Vascular Surgery	2025	Prospective Cohort	Japan, Asia	1 year	12/2021-02/2024	⊕⊕○○Low
Willems et al.	Annals Of Vascular Surgery	2024	Retrospective Cohort	Department of Vascular Surgery, Leiden University Medical Centre, Leiden, The Netherland, Europe	3 months and 1 year	Data from 01/01/2006-01/01/2022	⊕⊕○○Low
Yagyu et al.	Japanese Circulation Journal	2020	Retrospective Cross-Sectional	National Cerebral and Cardiovascular Centre in Suita, Japan, Asia	No	01/2015-12/2017	⊕⊕○○Low

GRADE: Grading of Recommendations, Assessment, Development, and Evaluation

**Table 2:** Patient demographics, comorbidities, and medical therapy across included studies

Authors	Sample Size	No. Interventions	Mean Age (years)	Male *n*	AHT *n*	Dyslipid *n*	DM *n*	Smoking *n*	CAD *n*	CKD *n*	Statins *n*	Antiplatelet Therapy *n* (%)	Anticoagulation *n* (%)
Arévalo-Zamora et al.	68	130	NA	33	57	37	34	33	No	13	NA	NA	NA
de Castro-Santos et al.	141	198	69	78	120	49	74	85	31	NA	49	NA	NA
Del Valle et al.	100	199	66	60	35	19	27	19	NA	NA	NA	NA	NA
Hart et al.	265	307	71 (median)	168	NA	NA	196	NA	NA	53 ESRD	NA	NA	NA
Junaidi et al.	58	58	60,2	27	NA	NA	38	NA	NA	NA	NA	NA	NA
Mendes-Pinto et al.	55	110	74,5	32	NA	NA	46	NA	NA	NA	NA	NA	NA
Ochoa-Ayón et al.	31	31	67,29	26	28	NA	19	NA	NA	21	NA	NA	NA
Pinelo et al.	53	60	73,4	38	NA	NA	53	NA	NA	NA	NA	NA	NA
Silva et al.	88	100	75 (median)	71	75	67	88	24	27	18 ESRD	NA	NA	NA
Sommerset et al.	188	227	70	131	130	119	81	111	77	6	NA	NA	NA
Spiliopoulos et al.	28	28 Limbs, 68 Vessels	NA	21	21	18	22	10	14	11 ESRD	NA	NA	NA
Teso et al.	72	73	Limb Salvage: 73,1; Limb Loss: 75,3	Limb Salvage: 26; Limb Loss: 3	Limb Salvage: 53; Limb Loss: 14	NA	Limb Salvage: 56 Limb Loss: 14	Limb Salvage: 27 Limb Loss: 9	Limb Salvage: 32 Limb Loss: 10	Limb Salvage: 14 Limb Loss: 12 ESRD	NA	NA	NA
Trihan et al.	137	258	70 (median)	86	94	65	60	69	63	22 ESRD	NA	NA	NA
Trihan et al.	77	68	69	57	63	50	22	37	20 (MI and/or Stroke)	11 ESRD	41	60	NA
Tun et al.	41	51	70,9	30	39	32	31	23	23	24 (10 on dialysis)	37	52	15
Willems et al.	136	143 wounds	70,1	92	95	77	64	47	75	30 (GFR < 60)	NA	NA	NA
Yagyu et al.	211	270	72,1	163	170	128	91	183	68	25 ESRD	NA	NA	NA

AHT: Arterial Hypertension; CAD: Coronary Artery Disease; DM: Diabetes Mellitus; ESRD: End Stage Renal Disease (EGFR<30mL/min); GFR: Glomerular Filtration Rate; NA: Not Available; MI: Myocardial Infarction

PAT methodology data included analysed arteries, PAT protocols with ABI correlated categories, comparative perfusion assessments including ABI and transcutaneous oxygen pressure (TcPO2), clinical staging systems, such as Wound, Ischaemia, and foot Infection Classification (WIfI) and Rutherford (when available), and pre- and post-operative PAT (when available). Extracted outcomes included wound healing rates at short-term (30 days) and long-term (3–12 months) follow-up, major amputation incidence, and corresponding PAT value and wound healing threshold PAT values. Data from the diagnostic accuracy of PAT compared to ABI were extracted to enable meta-analysis of the primary endpoint. The completed data extraction sheet is provided in Tables 3 and 4. Given the heterogeneity in study populations and analytical methodologies, information on the covariates considered for confounding adjustment in the original studies is reported in Supplemental Table 3, while study-specific inclusion and exclusion criteria are detailed in Supplemental Table 4. Both are available in Additional File 1.

**Table 3:** Clinical classification systems used across included studies, diagnostic thresholds, protocols, and accuracy metrics of PAT

Authors	WIfI / Rutherford Classification	Wound Healing Threshold	PAT Protocol	Comparative Assessment tool	Analysed Artery	Sensitivity (Sens.), Specificity (Spec.) and NPV	AUC
Arévalo-Zamora et al.	NA	PAT>160ms for patients with an ABI < 0.5 PAT<100ms for normal ABI	Group1: PAT<120ms (normal ABI); Group 2: PAT: 120–160ms, (borderline ABI and ABI 0.5–0.9); Group 3: PAT>160ms (ABI < 0.5)	ABI	Lateral, medial artery and deep plantar arteries	For cut-off > 160ms: Sens: 92.3%; Spec: 83,7%; NPV: 98,9% For cut-off < 100ms: Sens: 70,7%; Spec: 81,2%; NPV: 86,5%	For cut-off > 160ms: 0,89
de Castro-Santos et al.	WIfI	204ms to detect ABI < 0.4 (WIfI grade 3)	3 ABI groups: ABI < 0.8; ABI < 0.6, and ABI < 0.4; 2 classes based on their WIfI stages: 1 and 2	ABI	Lateral plantar artery	Patients with diabetes: Sens: 65%; Spec: 88%; NPV: 90%; Patients without diabetes: Sens: 74%; Spec: 90%; NPV: 90%	Patients with and without diabetes AUC: 0.90
Del Valle et al.	NA	PTA: multilevel disease, 182ms. ATA: multilevel disease 194ms	NA	Not used	Distal segments of posterior and anterior tibial arteries	No	No
Hart et al.	WIfI	Wound healing: 106ms; AFS: 104ms	Cat.1 (asymptomatic, 20- 120ms); Cat.2 (mild ischaemia, 121-180ms); Cat.3 (moderate ischaemia; 181-224ms); Cat.4 (CLTI>225ms)	Not used	Arcuate, first dorsal metatarsal, medial and lateral plantar, and deep plantar arteries	No	No
Junaidi et al.	NA	No	NA	DUS	Popliteal, anterior and posterior tibial arteries	No	No
Mendes-Pinto et al.	Rutherford	No	NA	Arterial stiffness indexes, PWV, ABI, peripheral SBP, DBP, central SBP, DBP, central and peripheral PP, augmentation index for HR of 75 bpm	Dorsal pedal artery, posterior tibial artery, and plantar arteries	No	No
Ochoa-Ayón et al.	WIfI	Reduction of PAT to class 1 or 2 allows the prediction of limb salvage in patients revascularised by angioplasty	Cat.1 (asymptomatic, 20-120ms; ABI: 1.3–0.90); Cat.2 (mild ischaemia, 121-180ms; ABI: 0.89 − 0.69); Cat.3 (moderate ischaemia, 181-224ms; ABI: 0,68 − 0,50); Cat.4 (CLTI>225ms; ABI: 0.40-0,00)	Not used	Arcuate and dorsal metatarsal arteries; medial, lateral, and deep plantar arteries	No	No
Pinelo et al.	WIfI	PAT > 186ms	NA	TcPO2	Dorsal metatarsal, lateral and medial plantar, branches from posterior tibial or peroneal arteries; collateral flow	Sens: 50%; Spec: 96%	0.774
Silva et al.	NA	180,5ms	HW- mild ischaemia, Cat.2 (PAT 121-180ms); NHW – moderate ischaemia, Cat.3 (PAT 181-224ms); revascularised and amputated – severe ischaemia, Cat.4 (PAT>225ms)	Not used	Most distal foot artery with adequate Doppler sign, supplying the wound area: plantar, dorsal pedis, arcuate, deep plantar	Sens: NA; Spec: 81%; NPV: NA	0,72
Sommerset et al.	TASC classification	Values > 120ms were considered indicative of PAD	NA	ABI and TP	Lateral, medial and deep plantar arteries, dorsal metatarsal artery, arcuate artery	When PAT > 0: Worst case PAT: Sens: 71%; Spec: 79%; NPV: 66% Arteries: Lateral Plantar: Sens: 60%; Spec: 71%; NPV: 57% Medial Plantar: Sens: 54%; Spec: 83%; NPV: 56% Dorsal Metatarsal: Sens: 71%; Spec: 70%; NPV: 77% Arcuate: Sens: 58%; Spec:73%; NPV: 56% Deep Plantar: Sens: 57%; Spec: 83%; NPV: 59%	When PAT > 0: Worst case PAT:79%; Arteries: Lateral plantar:72%; Medial plantar: 72%; Dorsal metatarsal: 77%; Arcuate:71%; Deep Plantar:73%
Spiliopoulos et al.	WIfI	Post procedural PAT < 120ms correlates with wound healing after 6 months	NA	PI	Dorsalis pedis and plantar arteries; if not possible, distal posterior tibial arteries	No	No
Teso et al.	WIfI	A change in PAT from class 4 to 2 is an independent predictor for limb salvage	Cat.1 (asymptomatic, 20-120ms; ABI: 1.3–0.90); Cat.2 (mild ischaemia, 121-180ms; ABI: 0.89 − 0.69); Cat.3 (moderate ischaemia, 181-224ms; ABI: 0,68 − 0,50); Cat.4 (CLTI > 225ms; ABI: 0.40-0,00)	ABI	Ant. circulation: arcuate and first dorsal metatarsal arteries, Post. circulation: medial, lateral, and deep plantar arteries	No	No
Trihan et al.	NA	AT_max_ of 215ms to detect CLTI	NA	ABI, TBI, TP	Dorsal pedal and lateral plantar arteries	To diagnose CLTI: Sens: 95%; Spec: 92%; NPV: 100%	0,98 (for a cut-off of 213ms)
Trihan et al.	NA	AT_max_ of 215ms to detect a TP of 30mmHg	NA	TP, ABI, TBI, PSV, EDV, RI	Dorsal pedal and lateral plantar arteries	Sens: 86%; Spec: 81%; NPV: 97%	0,89
Tun et al.	WIfI	No; PAT correlated anatomically with wound location to arcuate and deep plantar arteries (mean PAT 156ms) and dorsal metatarsal and medial plantar (mean PAT 165ms)	Cat.1 (asymptomatic, 20-120ms); Cat.2 (mild ischaemia, 121-180ms); Cat.3 (moderate ischaemia; 181-224ms); Cat.4 (CLTI>225ms)	ABI; TP	Dorsal pedal, lateral, medial, deep plantar, arcuate and dorsal metatarsal arteries	No	No
Willems et al.	Wagner Classification	ACC_max_ <1.0 m/s^2^ Associated with nonhealing ulcer	Cat.1 (no ischaemia,>5,5 m/s^2^), Cat.2 (mild ischaemia, 3.0–5,5 m/s^2^), Cat.3 (moderate ischaemia, 1,0–3,0 m/s^2^), Cat.4 (severe ischaemia < 1,0 m/s^2^)	Not used	Digital artery of hallux and dorsal pedal artery	For ACC_max_<1,0 m/s^2^ 3 months: Sens: 57,1%; Spec: 85,3%; NPV: 94,8%; 12 months: Sens: 44,4%; Spec:89,7%; NPV: 82,8% For ACC_max_<0,5 m/s^2^ 3 months: Sens: 85,7%; Spec: 73,6%; NPV: 97,9%; 12months: Sens: 61,1%; Spec: 77,6%; NPV: 85,6%	No
Yagyu et al.	Rutherford	Prediction of aortoiliac lesion: 103ms; Prediction of femoropopliteal lesion: 105ms	NA	PSV and Spectral waveform Doppler Contour	Common femoral artery and popliteal artery	For aortoiliac lesions: Sens: 82%; Spec: 76%; NPV: 75% For femoropopliteal lesions: Sens: 86%; Spec: 67%; NPV: 75%	No

ABI: Ankle Brachial Index; ACC_max_: Maximal systolic acceleration; AFS: Amputation Free Survival; ATA: Anterior Tibial Artery; AT_max_: maximal acceleration time; AUC: Area Under the Curve; CLTI: Chronic Limb-Threatening Ischaemia; DBP: Diastolic Blood Pressure; DUS – Doppler Ultrasound; EDV: End Diastolic Velocity; HR: Heart Rate; HW: Wound Healing; NA – Not Available; NPV: Negative Predictive Value; NWH: Non-Wound Healing; PAT: Pedal Acceleration Time; PI: pulsatility Index; PP: Pulse Pressure; PSV: Peak Systolic Velocity; PTA: Posterior Tibial Artery; PWV: Pulse Wave Velocity; RI: Resistivity Index; SBP: Systolic Blood Pressure; TASC grade: Transatlantic Inter-society Consensus; TBI: Toe Brachial Index; TP: Toe Pressure; WIfI: Wound Ischaemia Foot Infection classification

**Table 4:** Post-interventional pedal acceleration time and limb-related outcomes

Authors	Post-interventional PAT	Major Lower-Limb Amputation	Short-Term Wound Healing (30 days)	Long-Term Wound Healing (3–12 months)	All-Cause Mortality
Arévalo-Zamora et al.	No	No	No	No	No
de Castro-Santos et al.	No	No	No	No	No
Del Valle et al.	No	No	No	No	No
Hart et al.	No	AFS patients: PAT: 104ms vs. amputated: PAT: 136ms	No	Yes − 1Y proportion of wound healing decreased as the final PAT increased	Yes − 1Y - Patients who died (93): PAT: 144ms vs. patients alive (214): PAT: 108ms
Junaidi et al.	No	No	No	Status following BKA (below knee amputation), 31 healed; 27 non-healed*	No
Mendes-Pinto. et al.	No	No	No	No	No
Ochoa-Ayón et al.	Pre-angioplasty PAT: 213ms (± 83.26); Post-procedure, PAT: 118ms (± 16.40); Average PAT decrease 94.64ms (± 72.87)	No	Yes − 30 patients	1 wound not healed*	No
Pinelo et al.	Initial patient evaluation:135 ± 6ms for patients achieving successful wound healing and 190 ± 12ms for the failing-to-heal patients	Yes- 4 patients (culminated in major amputation PAT>186ms in all of them)	No	No	No
Silva et al.	Mean PAT in HW: 159.8ms, NHW: 199.32ms, revascularised: 239.18ms (amputated), 279.1ms	Yes- mean PAT: 279.14ms	Mean PAT in each group was 159.81ms (HW), 199.32ms (NHW), 239.18ms	3-6 M NHW: wounds with a positive healing process: mean PAT 189.96ms vs. 231.43ms in worse evolution wounds	No
Sommerset et al.	No	No	No	No	No
Spiliopoulos et al.	Preprocedural PAT: 154.15 ± 70.35ms; postprocedural to 107.21 ± 49.6ms	Yes – No amputation PAT values available	No	Yes – No PAT values available	Yes- No PAT values available
Teso et al.	PAT pre-procedure: 224 ± 25ms; post-procedure: 144 ± 49ms	Yes–14 limbs. PAT pre-procedure 244 ± 30ms; post-procedure 213 ± 47ms	No	No	No
Trihan et al.	No	No	No	No	No
Trihan et al.	No	No	No	No	No
Tun G. et al.	No	1Y follow up (8patients) No significant link between highest PAT and amputation	No	No	No
Willems et al.	No	Yes- ACC_max_ <1 m/s^2^ associated with higher amputation risk At 12 M: mean ACC_max_ at hallux 0,28 m/s^2^; at dorsal pedal artery 0,41 m/s^2^	14 healed ulcers after 3 M	24 healed ulcers after 6 M;36 after 12 M For 12 M: mean ACC_max_ at hallux 1,44 m/s^2^; at dorsal pedal artery 2,89 m/s^2^	No
Yagyu et al.	No	No	No	No	No

ACC_max_: Maximal systolic Acceleration; AFS: Amputation Free Survival; BKA: Below the Knee Amputation; HW: Wound Healing; M: Month(s); NWH: Non-Wound Healing; PAT: Pedal Acceleration Time; Y: year

*No Pedal acceleration time reported

### Assessment of study quality

Concerning qualitative assessment, the National Heart, Lung, and Blood Institute (NHLBI) Study Quality Assessment Tool for observational cohort and cross-sectional studies (2021) was used [17]. This assessment was independently conducted by two authors (MRF and ES), and when disagreements arose, decisions were reached by mutual consensus following a third-party review (JRN). The quality of evidence for the included articles was evaluated using the Grading of Recommendations, Assessment, Development, and Evaluation (GRADE) approach [18]. Articles were classified into four levels of quality (high, moderate, low, and very low).

### Quantitative synthesis

A diagnostic test accuracy meta-analysis using a bivariate random-effects meta-analysis approach was performed. This method is equivalent to the hierarchical summary receiver operating characteristic model and overcomes many of the limitations of non-hierarchical models, and allows for the calculation of summary properties of diagnostic tests (such as pooled sensitivity, specificity and area under the receiving operating characteristic [ROC] curve), with the advantage of jointly estimating sensitivity and specificity, taking into account their correlation [19, 20]. For studies reporting ABI as the comparator, ABI-defined ischemia was treated as the reference standard, recognising that ABI is an imperfect surrogate in patients with incompressible vessels. A correlation coefficient (*r*) > 0.6 between sensitivity and false positive rate was considered a considerable threshold effect (i.e., negative correlation between sensitivity and specificity possibility accounted for by cutoff variability) [21, 22].

Heterogeneity was evaluated through the I^2^ Q statistic-related measure of variance, and a diagnostics random-effects model was preferred to obtain summary sensitivity, specificity, positive likelihood ratio (PLR), negative likelihood ratio (NLR), and diagnostic odds ratio (DOR). Forest plots were used to display results from the meta-analysis. Sources of heterogeneity were assessed by leave-one-out sensitivity meta-analysis. Assessed covariates included the publication year, participants’ mean age, percentage of male participants, percentage of patients with arterial hypertension, dyslipidaemia, diabetes, or coronary artery disease, percentage of chronic kidney disease, and use of antiplatelet therapy.

Publication bias was assessed using funnel plots. Multivariable meta-regression analysis was not possible to perform because of an insufficient number of primary studies (< 10). In addition, a leave-one-out sensitivity meta-analysis was conducted to evaluate the robustness of the pooled estimates and assess the influence of individual studies on the overall results, excluding each study in turn.

All statistical analyses were performed using Open Meta^®^ (MetaMorph, Inc., Menlo Park, California, USA).

## Results

### Search results

**Fig. 1:**
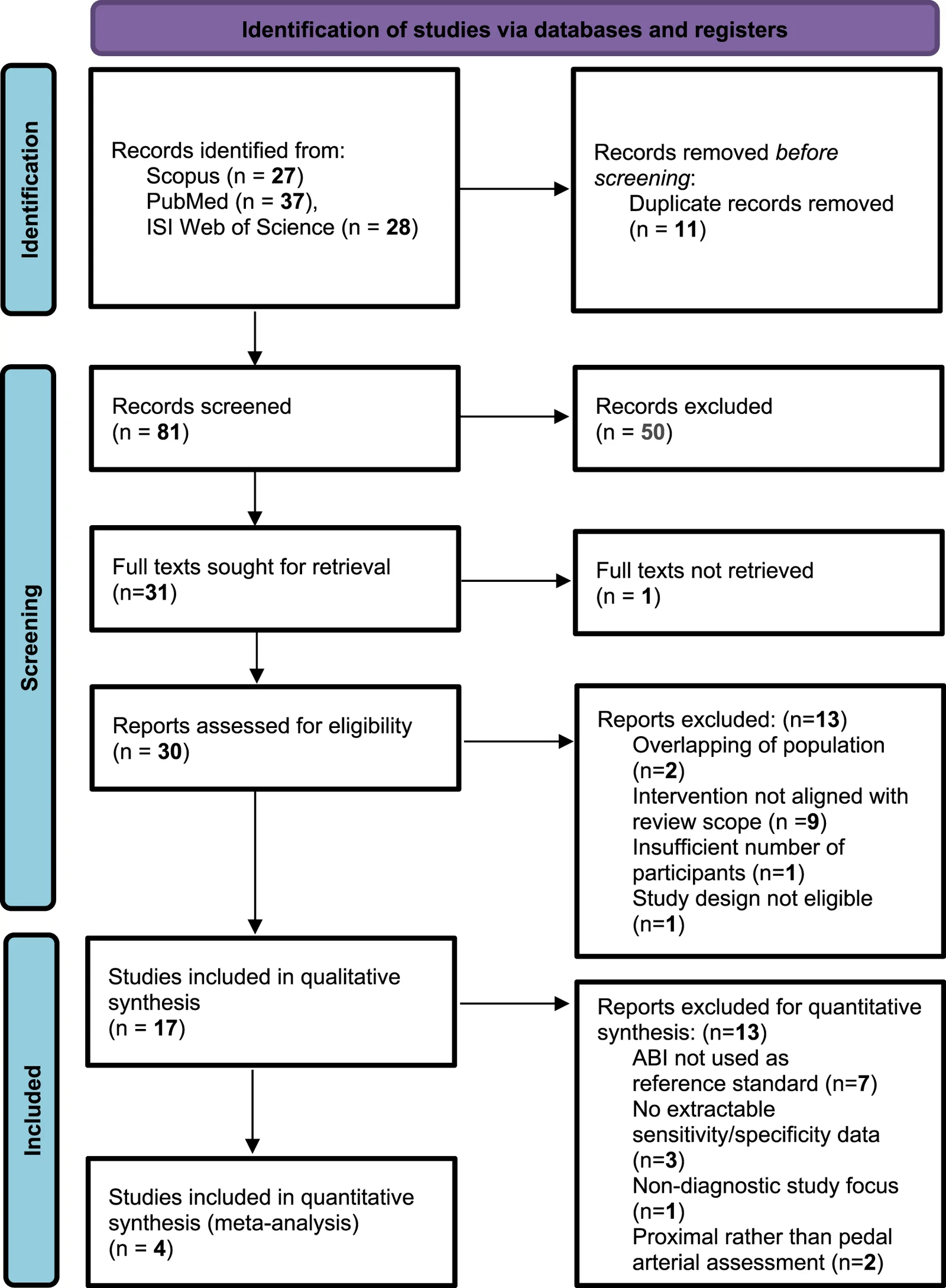
PRISMA 2020 flow diagram for new systematic reviews which included searchers of databases and registers only. Page MJ, McKenzie JE, Bossuyt PM, Boutron I, Hoffmann TC, Mulrow CD, et al. The PRISMA 2020 statement: an updated guideline for reporting systematic reviews. BMJ 2021;372:n71. doi: https://doi.org/10.1136/bmj.n71. For more information, visit: http://www.prisma-statement.org/

After the database search and duplicate exclusion, a total of 81 studies were screened. Upon title and abstract screening, 50 studies were excluded. For full-text assessment, 31 studies were eligible; 13 were excluded, and 1 was not retrieved (Fig. 1). The criteria for exclusion upon full-text assessment were repeated patient population (*N* = 2), intervention not aligned with review scope (*N* = 9), insufficient number of participants (*N* = 1) and study design not eligible (*N* = 1). Thus, a total of 17 published articles were included in this systematic review. No studies were excluded from the systematic review or meta-analysis solely on the basis of their quality assessment score.

### Description of studies

Among the 17 studies included in this systematic review, 8 were cross-sectional [11, 14, 23–28], 1 was a prospective observational study [29], and other 3 were retrospective observational [9, 12, 13]. There were also 4 cohorts [30–33], 3 of them being prospective [30, 32, 33], 1 retrospective [31] and 1 case-control study [34]. The included publications reported studies performed in 14 different countries within 5 continents – 5 from North America [9, 25, 27, 32, 33], 3 from South America [11, 14, 24], 6 from Europe [12, 13, 23, 26, 29, 31], 2 studies from Asia [28, 34] and 1 from Oceania (30). One study was conducted across both North America and Oceania [27]. A total of 1749 patients were assessed, with a minimum of 28 and a maximum of 265 patients per study, and the mean participants’ age was 70.4 years. The percentage of male participants was 65,3% (*n* = 1142). Demographics and comorbidities of the populations included in the studies are summarised in Table 2.

### Main findings and meta-analysis

Despite heterogeneity in prognostic outcome reporting, the protocol used to measure PAT was largely consistent across studies, with comparable Doppler acquisition techniques. This methodological consistency was primarily reflected in the anatomical level and arterial targets selected for PAT assessment. Across the included studies, PAT was consistently measured in distal pedal arteries below the ankle, predominantly involving the dorsal pedal system (dorsal pedis, dorsal metatarsal, and arcuate arteries) and/or the plantar circulation (including the medial, lateral and deep plantar arteries), which correspond to the distal arterial perfusion of the foot. In several studies, PAT was assessed in the most distal artery with an adequate Doppler signal supplying the wound area, thereby linking PAT assessment to functionally relevant perfusion territories. In addition, PAT was predominantly analysed as a categorical rather than a continuous variable. Most studies stratified PAT values into classes reflecting increasing ischaemia severity, using predefined thresholds. Several stratification schemes were based on the originally described stratification scheme by Sommerset et al. [35], while other studies applied conceptually similar categorical frameworks, in some cases explicitly aligned with ABI-defined ischaemia severity.

Of the 17 studies included in the systematic review, only 4 provided data suitable for quantitative diagnostic meta-analysis. The remaining 13 studies were excluded from pooled analysis because they did not report paired sensitivity and specificity estimates against a standardised reference standard, did not use ABI as a formal comparator in a format permitting 2 × 2 contingency table construction, reported PAT exclusively as a continuous or categorical prognostic variable, or assessed acceleration parameters at proximal rather than pedal arterial levels. This selection criterion was applied to ensure methodological homogeneity of the pooled diagnostic estimates.

**Fig. 2:**
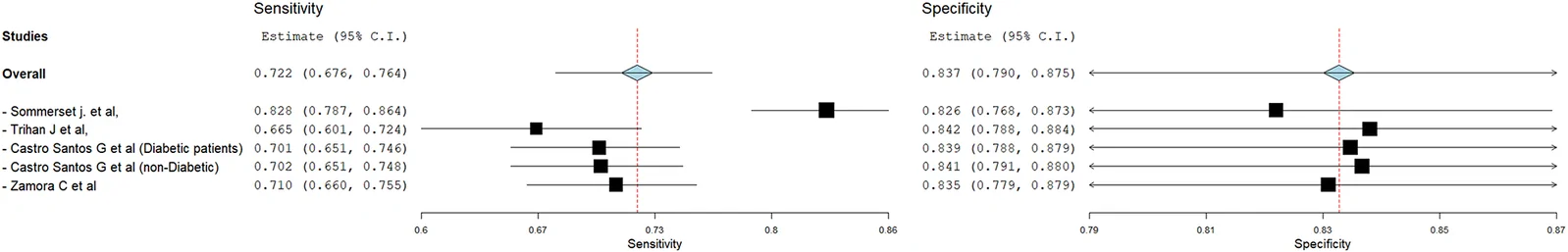
Pooled sensitivity and specificity of PAT for detecting clinically significant lower-limb ischaemia. Forest plots showing pooled and study-specific sensitivity and specificity estimates of PAT for detecting clinically significant lower-limb ischemia, using ABI-defined ischemia, with 95% confidence intervals

In total, 4 primary studies reported extractable diagnostic data, suitable for quantitative synthesis [10–12, 14]. One study [14] reported separate estimates for diabetic and non-diabetic subgroups, yielding five analytical datasets. Across these datasets, pooled sensitivity of PAT for detecting clinically significant lower-limb ischaemia, according to ABI-based definitions, was 0.72 (95% CI 0.68–0.76), with individual study estimates ranging from 0.66 to 0.83 (Fig. 2). Specificity was consistently high across cohorts, yielding a pooled value of 0.84 (95% CI 0.79–0.88). Between-dataset heterogeneity for both parameters was minimal (Fig. 2).

**Fig. 3:**
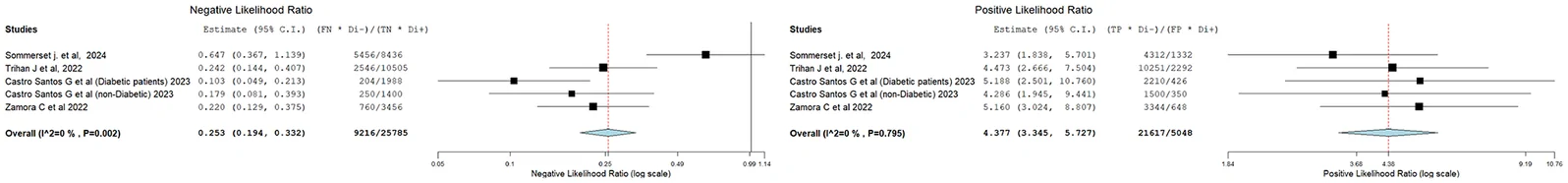
Positive and negative likelihood ratios of PAT for detecting lower-limb ischaemia. Forest plots showing pooled and study-specific positive and negative likelihood ratios of PAT for detecting clinically significant lower-limb ischemia, with 95% confidence intervals

The pooled PLR was 4.38 (95% CI 3.35–5.73), indicating that higher PAT values were associated with a meaningful increase in the post-test probability of significant ischaemia. Correspondingly, the NLR was 0.25 (95% CI 0.19–0.33), demonstrating that lower PAT values were associated with a substantial reduction in the likelihood of clinically significant limb ischaemia. Both PLR and NLR showed low heterogeneity (I² = 0%), supporting consistency across diverse patient groups, including diabetic and non-diabetic subpopulations (Fig. 3). The I² value of 0% for positive and negative likelihood ratios indicates negligible between-study heterogeneity, consistent with the physiological stability of the PAT measurement across diverse patient populations and acquisition settings.

**Fig. 4:**
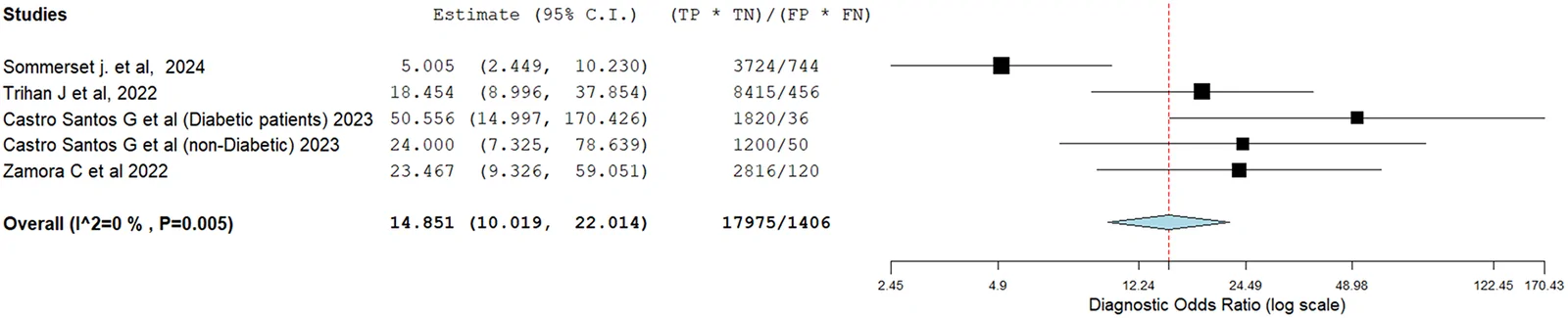
Diagnostic odds ratio of PAT for detecting lower-limb ischaemia. Forest plot showing pooled and study-specific diagnostic odds ratio estimates of PAT for the detection of clinically significant lower-limb ischemia, with 95% confidence intervals, reflecting the overall discriminatory performance of PAT across studies

The combined DOR was 14.85 (95% CI 10.02–22.01), reflecting strong overall diagnostic discriminatory performance. Individual study DORs varied substantially, ranging from 5.01 to 50.56, which is expected given the differences between the population’s profiles and the prevalence of advanced PAD. However, the pooled estimate remained stable under random-effects modelling (Fig. 4). To assess the robustness of the pooled estimates and the potential influence of individual datasets, leave-one-out sensitivity analyses were conducted. Sequential omission of each dataset did not result in any material change in the pooled diagnostic indices, supporting the stability of the meta-analysis findings despite differences in their relative contributions.

**Fig. 5:**
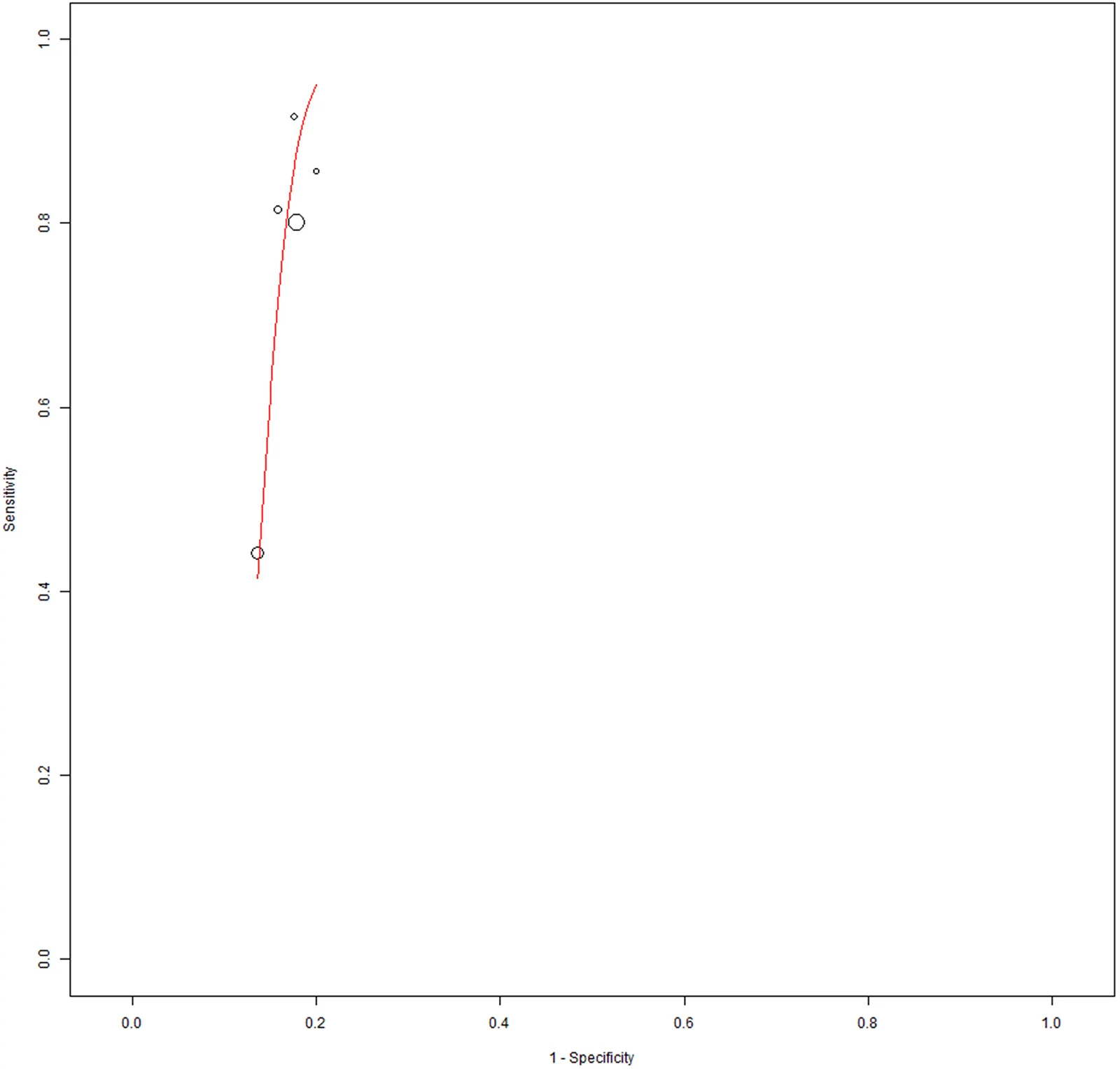
SROC of PAT for detecting lower-limb ischaemia. SROC illustrating the overall diagnostic performance of PAT for detecting clinically significant lower-limb ischaemia. Each point represents an individual study estimate, and the fitted curve summarises the trade-off between sensitivity and specificity across included databases

The summary ROC (SROC) curve demonstrated strong overall diagnostic performance of PAT in identifying clinically significant lower-limb ischaemia. The individual study estimates clustered toward the upper left quadrant of the plot, indicating consistently high specificity and moderate-to-high sensitivity across datasets. The fitted SROC curve showed a steep ascent with minimal dispersion, reflecting low between-study variability and supporting the robustness of the pooled diagnostic estimates. Although the number of available studies was reduced, the SROC shape indicates that PAT maintains a reliable capacity of discrimination across different thresholds and patient profiles (Fig. 5).

Due to insufficient standardisation and incomplete reporting of wound healing outcomes across the included studies, a pooled meta-analysis evaluating the prognostic value of PAT for wound healing was not feasible. As a result, findings related to wound healing were summarised qualitatively. Across the included studies reporting wound healing outcomes, patient populations predominantly consisted of individuals with advanced peripheral artery disease, most frequently in the context of chronic limb-threatening ischaemia (CLTI).

### Studies quality

**Fig. 6:**
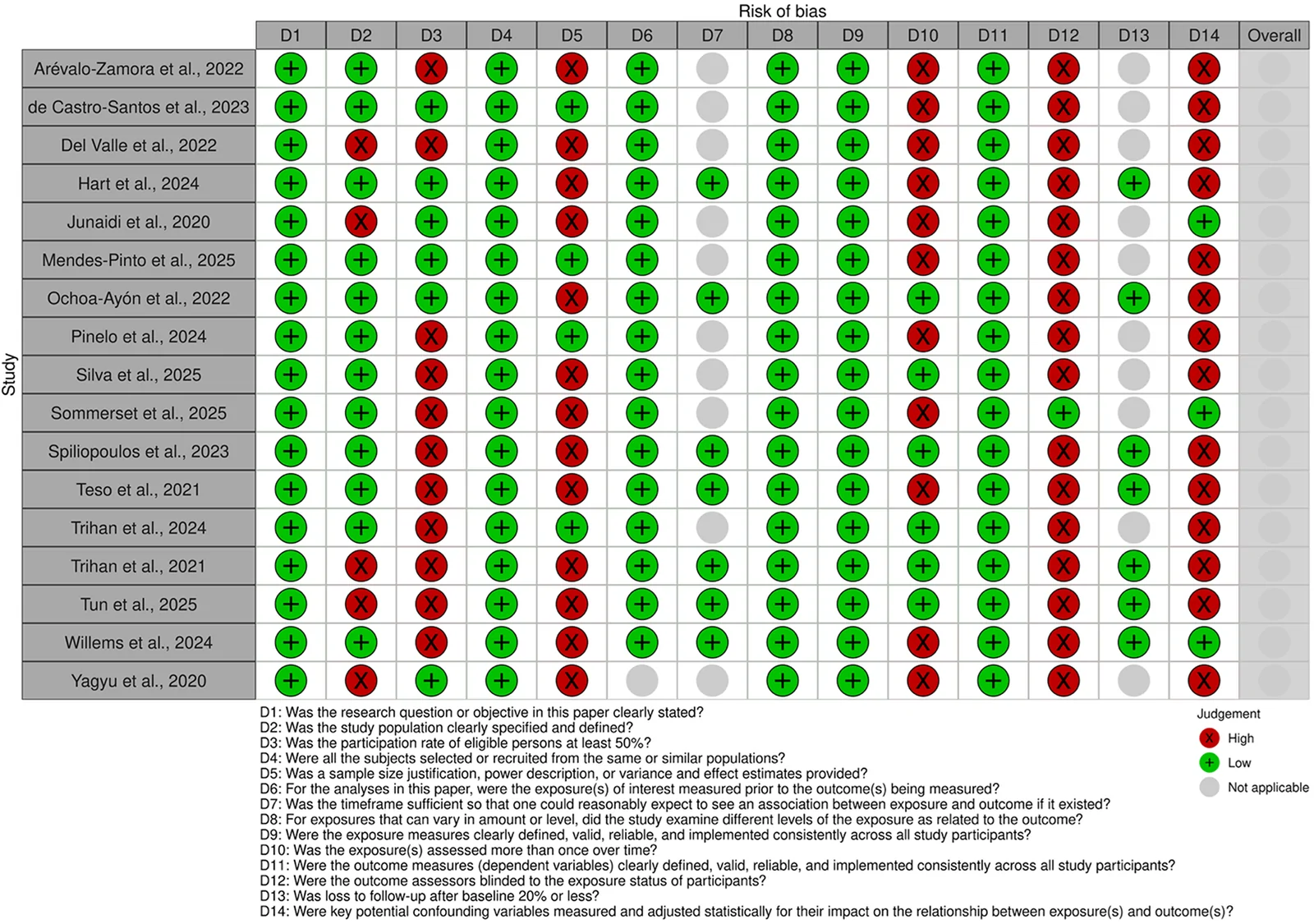
Risk of bias of included studies using the NHLBI Study Quality Assessment Tool

**Fig. 7:**
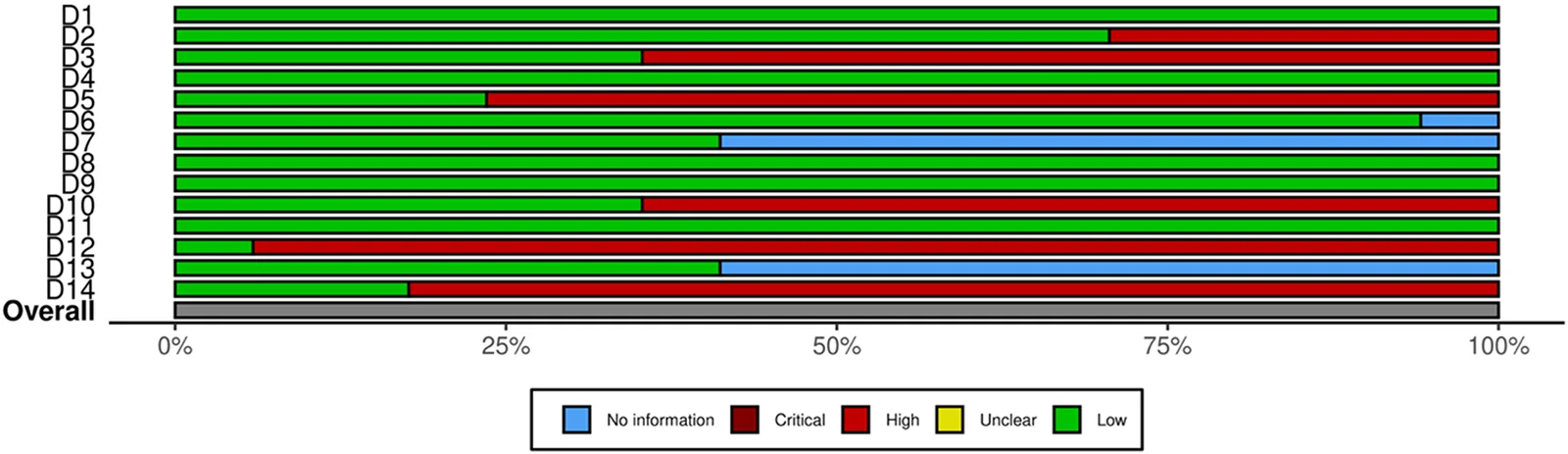
Risk of bias of included studies by item, using the NHLBI Study Quality Assessment Tool

The risk of bias in the selected articles is displayed in Fig. 6. The overall judgement, per evaluated item, across the included observational studies is shown in Fig. 7.

Across the included studies, the overall methodological quality was moderate, with substantial variability in specific domains. Most studies demonstrated a low risk of bias for clearly stated research objectives (D1), well-defined study populations (D2), appropriate participant recruitment (D4), and adequate exposure and outcome measurement (D8–D11). However, several recurrent limitations were identified. A notable proportion of the studies showed a high risk of bias in participation rates (D3) and a lack of sample size justification or power calculation (D5), reflecting limited methodological transparency. Additionally, confounding control (D14) was frequently inadequate, with many studies failing to adjust for key variables (e.g., calcification, renal replacement therapy), thereby increasing the potential for residual confounding by unaccounted or unmeasured factors. Follow-up adequacy (D13) when applicable, was generally well controlled and did not represent a major source of bias across the included studies.

### Publication bias

A funnel plot could not be constructed because the number of included studies was insufficient for meaningful assessment of publication bias. Consequently, the presence of publication bias cannot be ruled out.

## Discussion

In this systematic review and meta-analysis comparing PAT with ABI, PAT demonstrated consistent diagnostic performance and robust discriminatory ability for detecting clinically significant lower-limb ischaemia. PAT showed high specificity, robust likelihood ratios, and a diagnostic odds ratio close to 15, supporting reliable identification of ischaemia, including in clinical contexts where ABI measurements may appear normal or be unreliable. The pooled diagnostic indices yielded a sensitivity of 0.72 (95% CI 0.68–0.76), a specificity of 0.84 (95% CI 0.79–0.88), a PLR of 4.38 (95% CI 3.35–5.73), an NLR of 0.25 (95% CI 0.19–0.33), and a DOR of 14.85 (95% CI 10.02–22.01), supporting a strong association between PAT measurements, ABI-defined ischaemia, and clinically relevant lower-limb perfusion deficits.

### Diagnostic performance of PAT

Additional findings from the included studies strengthen the physiological reliability of PAT and further clarify its relationship with established perfusion metrics. Arévalo-Zamora et al. [11], in a cross-sectional study with 68 patients, demonstrated a clear haemodynamic continuum across ABI strata, with mean PAT in the lateral plantar and arcuate arteries rising from short values (< 120ms) in limbs with normal ABI (1.0-1.3), through intermediate values borderline and mild to moderate disease (ABI 0.50–0.89), to markedly prolonged values (> 160ms) in severe disease (ABI ≤ 0.50), thereby linking progressive prolongation of PAT to increasingly impaired macrovascular perfusion. Similarly, in patients with CLTI and non-compressible arteries, Teso et al. [9], in an observational study with 73 patients, showed that higher PAT classes were predominantly observed in limbs that progressed to major amputation, whereas lower PAT classes were associated with limb salvage, indicating that PAT preserves prognostic discrimination even when ABI cannot be reliably interpreted. In a cross-sectional study including 141 patients (198 limbs), de Castro-Santos et al. [14] further demonstrated that PAT closely reflected ABI-defined ischaemia severity. A PAT cut-off ≥ 96ms detected ABI < 0.80, while a higher cut-off ≥ 204ms identified ABI < 0.4 with 88% accuracy in diabetic patients and 87% in non-diabetic patients.

### PAT in special populations

Notably, these diagnosis and staging relationships were preserved with diabetic and chronic kidney disease populations, where ABI is known to potentially underestimate disease severity, with selected PAT cut-offs achieving sensitivities up to 98% and specificities above 80%, as shown in the study by de Castro-Santos et al. [14] and Trihan et al. [12]. In parallel, Pinelo et al. [23] and Spiliopoulos et al. [29], reported concordance between prolonged PAT, low TcPO2, and impaired wound healing, positioning PAT as a particularly robust perfusion marker in groups of patients characterised by medial calcification, complex microvascular pathology, and high residual limb-threatening risk.

A more detailed examination of individual studies shows that PAT not only detects haemodynamically relevant ischaemia but also enhances clinically interpretable stratification of wound healing and limb salvage across baseline and post-revascularisation settings. Although PAT is a continuous Doppler-derived parameter, most studies have translated absolute values into categorical classes or clinically relevant thresholds, often aligned with the previously described Sommerset classification [34] to facilitate interpretation and stratification by ischaemic risk. In the cross-sectional study by Pinelo et al. [23], with 53 patients and 60 interventions, the mean post-operative PAT was 135ms in ulcers that healed versus 190ms in those that failed to heal. Values exceeding the threshold of 186ms were strongly correlated with limbs with a reduced probability of wound healing. Silva et al. [26] conducted a retrospective cross-sectional study evaluating 88 patients (100 ischaemic limbs) and confirmed a PAT wound healing cut-off of 180.5ms. In this study, healed wounds had a mean PAT of 159.8ms, whereas non-healed, revascularised, and amputated limbs showed progressively higher mean values (193.3ms, 239.2ms and 279.1ms, respectively), supporting a practical healing threshold near 180ms and reinforcing the prognostic value of PAT stratification in diabetic PAD. Mendes-Pinto et al. [24], in a cross-sectional study with 55 patients, and Hart et al. [30], who analysed a cohort of 265 patients and 307 limbs, likewise documented higher PAT values in patients with CLTI and elevated WIfI stages, supporting that values below approximately 180ms are indicative of adequate distal perfusion and favourable prognosis, while values in the 180-220ms range or higher delineate advanced ischaemia and substantially elevated risk of non-healing and limb loss. Sommerset et al. [27], with a cross-sectional study with 188 patients and 227 interventions, and Tun et al. [32] in a cohort with 41 patients and 51 interventions, reported similar associations between prolonged PAT and adverse limb outcomes in revascularised cohorts, reinforcing the consistency of these observations across different centres and groups of patients.

### Prognostic value for wound healing and amputation

Beyond wound healing, several studies indicate that PAT also stratifies major amputation risk in CLTI patients. Silva et al. [26] reported mean PAT values escalating from 159.8ms in healed wounds to 279.1ms in amputated limbs, supporting a practical cut-off near 225ms (PAT class 4, severe ischaemia) for identifying high amputation risk. Pinelo et al. [23] observed a PAT of 190 ± 12ms in failing-to-heal ulcers, culminating in major amputation, while Hart et al. [32] documented a PAT of 136ms in amputated vs. 104ms in amputation-free limbs at 1-year follow-up. Ochoa-Ayón et al. [33] and Teso et al. [9] further showed that failure to reduce PAT from class 4 (> 225ms, ABI < 0.4) to class 2 (121-180ms) post-revascularisation independently predicted amputation, despite technical success.

### Methodological considerations

A critical challenge for the clinical implementation of PAT is the absence of a universally accepted threshold. Across the included studies, cutoff values ranged from approximately 96ms for detecting moderate ischaemia (ABI < 0.80) to 225ms for defining severe or limb-threatening ischaemia, which not only reflects the continuous nature of this physiological parameter but also underlines the heterogeneity of reference standards and patient populations used for threshold derivation. Nevertheless, a degree of convergence is apparent in the literature. The categorical framework originally described by Sommerset et al. (normal (< 120ms), mild (121–180ms), moderate (181–224ms), and severe ischaemia (> 225ms))was adopted, explicitly or conceptually, by the majority of included studies, and the wound healing threshold of approximately 180ms was independently corroborated across multiple centres and patient profiles [27]. This convergence provides a pragmatic foundation for a standardised acquisition protocol.

Based on the collective evidence reviewed, the authors propose that future prospective studies adopt the following minimal standardisation framework: (1) PAT measured in the most distal pedal artery with an adequate Doppler signal supplying the wound territory; (2) categorical classification using the four-class Sommerset framework as a common reference [27]; (3) pre-specified thresholds of 120ms and 180ms as clinically meaningful decision points for ruling out significant ischaemia and predicting wound healing probability, respectively; and (4) parallel recording of ABI. Adoption of this framework in future multicentre registries would substantially accelerate the evidence base needed to establish PAT as a guideline-endorsed perfusion marker.

In cases where calcification or occlusion extends into the tarsal and plantar circulation, identifying an adequate Doppler signal may be technically challenging and operator-dependent. The pragmatic approach adopted across most included studies, measuring PAT in the most distal artery with an adequate signal supplying the wound territory, partially mitigates this limitation but does not eliminate it [26, 32]. No study reported a formal technical failure rate or quantified the proportion of patients in whom PAT could not be obtained, which reflects a significant gap in the current literature. Future prospective studies should prospectively record technical failure rates and operator experience levels to allow meaningful assessment of PAT feasibility across the full spectrum of CLTI severity.

Evidence from studies with longitudinal follow-up included supports an additional role for PAT in monitoring disease evolution and therapeutic response in CLTI. Ochoa-Ayón et al. [33] demonstrated that angioplasty was followed by a marked shift in PAT categories from predominantly moderate-severe ischaemia to mild or normal classes, closely correlating with limb preservation. In their series, mean PAT decreased from approximately 213ms pre-procedure (distributed across classes 2–4) to about 118ms post-procedure. Limbs achieving this reduction and transitioning into lower PAT categories (classes 1–2) were associated with favourable limb outcomes in infra-inguinal CLTI. Post interventional surveillance studies further refine this concept: Sommerset et al. [27], Tun et al. [32], Spiliopoulos et al. [29] and Willems et al. [31] reported that limbs with stable or improving PAT trajectories maintained wound stability and limb salvage, whereas rising or persistently abnormal PAT values, despite patent reconstructions, predicted the need for repeat revascularisation or major amputation, thereby supporting post-interventional PAT as a functional marker of clinical benefit following revascularisation.

Collectively, these findings support the integration of baseline and follow-up PAT into multidisciplinary CLTI pathways, alongside ABI, TcPO2 and imaging, as an objective ultrasound-based biomarker of revascularisation adequacy and limb salvage risk.

### Future clinical practice considerations

Notwithstanding variability in outcome reporting, measurement of pedal acceleration time (PAT) followed a largely standardised Doppler-based protocol across studies. Two methodological variations warrant consideration. First, in the study by Yagyu et al. [28], acceleration parameters were derived from femoral and popliteal arterial waveforms rather than from pedal arterial waveforms. Although conceptually related, proximal arterial acceleration reflects a different haemodynamic compartment and is not strictly equivalent to pedal acceleration time, which is intended to capture distal runoff and microvascular resistance. Second, Willems et al. [31] quantified maximal acceleration (ACC_max_) rather than time-to-peak acceleration (TAP/PAT). While both metrics describe systolic upstroke characteristics, ACC_max_ represents a velocity-derived amplitude parameter, whereas PAT is a time-based surrogate of distal perfusion, limiting direct interchangeability.

### Study limitations

This systematic review and meta-analysis, while providing comprehensive evidence on the diagnostic and prognostic value of PAT for lower-limb ischaemia, has several methodological constraints that must be considered. First, the number of eligible studies was limited, and sample sizes varied considerably, reducing statistical power and precluding subgroup or meta-regression analysis focused on specific arteries, acquisition settings, or reference standards. Nevertheless, the relatively narrow confidence intervals of pooled sensitivity, specificity, likelihood ratios, and diagnostic odds ratio, together with low to moderate heterogeneity, support the internal consistency of the summary estimates. Second, variability was observed across studies in the clinical application of PAT, including the arterial segments evaluated, the thresholds used to define abnormal values, and the reference standards applied for ischaemia classification. Although this variability may limit direct comparability and introduce spectrum or verification bias, most included studies enrolled patients presenting with clinical features such as non-healing ulcers or tissue loss, consistent with advanced ischaemia and CLTI. Importantly, higher PAT values were consistently associated with greater disease severity and poorer wound-related outcomes, suggesting a robust underlying physiological signal across diverse patient profiles, including those in whom ABI is less reliable. The use of ABI as the reference standard, while pragmatic and guideline-endorsed, introduces a risk of differential misclassification bias. In patients with incompressible vessels, ABI may underestimate ischaemia severity, leading to false-negative reference classifications that would artificially reduce PAT’s apparent sensitivity and potentially overestimate its specificity. This suggests that the pooled diagnostic accuracy estimates reported here may be conservative, and that PAT’s true discriminative performance, specifically in diabetic and calcified vessel populations, might exceed what the current meta-analysis captures. Furthermore, of the 17 studies included in the systematic review, only 4 provided data in a format suitable for quantitative diagnostic meta-analysis, as the remaining studies did not report paired sensitivity and specificity estimates against a standardised reference, precluding their inclusion in the pooled analysis. This discrepancy is a recognised methodological limitation of diagnostic test accuracy reviews and further underscores the need for standardised outcome reporting in future PAT studies. Evidence on prognostic performance for wound healing and limb outcomes was heterogeneous and could not be pooled. Finally, incomplete reporting of methodological aspects, such as blinding procedures and whether PAT thresholds were prespecified or derived from ROC analysis, may have introduced observer or selection bias. Other significant limitation of this meta-analysis is the small number of studies eligible for quantitative diagnostic synthesis. This substantially limits the generalisability of the pooled estimates, precludes meaningful subgroup analyses and meta-regression, and rendered formal funnel plot assessment of publication bias unfeasible. Few other short- and long-term outcomes were assessed, which deprived the study of PAT association, such as wound healing.

Future research should prioritise large, prospective, multicentre analysis of standardised PAT protocols, pre-specified thresholds, and clearly defined limb-based outcomes, directly comparing PAT with ABI, TcPO2, and other perfusion metrics. While ABI remains an established role in the initial evaluation of PAD, from the authors’ perspective, PAT offers particular advantages over ABI in four clinical scenarios: patients with diabetes mellitus and medial arterial calcification, where falsely elevated ABI values render pressure-based assessment unreliable; patients with chronic kidney disease or end-stage renal disease on dialysis, who share a similar calcification burden; patients with established CLTI and tissue loss requiring serial perfusion monitoring to assess revascularisation adequacy and guide wound management; and patients with incidentally identified non-compressible vessels (ABI > 1.40), in whom PAT should be considered a first-line alternative rather than a secondary investigation.

## Conclusion

This systematic review and meta-analysis suggest that PAT is a clinically valuable adjunct parameter for assessing limb perfusion and predicting wound-related outcomes in patients with PAD, particularly in advanced stages. PAT demonstrated solid diagnostic performance for clinically significant ischaemia, with a pooled sensitivity of 0.72, a specificity of 0.84, and a high diagnostic odds ratio of 14.9, indicating strong discriminative ability.

Despite the relatively limited number of available studies and variability in acquisition protocols and approaches for categorising PAT values, the internal consistency of pooled estimates and the concordance of findings across different centres, patient profiles, and comparator tests support PAT as a robust haemodynamic marker with clear clinical applicability. Across the included studies, lower PAT values were consistently associated with higher ABI measurements and more favourable wound healing outcomes, while prolonged PAT values were associated with impaired wound healing and increased amputation risk. As pooled prognostic analysis was not feasible, these associations are based on narrative synthesis of heterogeneous observational data and should be interpreted as hypothesis-generating rather than confirmatory.

Collectively, this work provides a strong rationale for incorporating PAT into multiparametric assessment of PAD and CLTI, alongside established perfusion tools, and underscores the need for larger, prospective, multicentre registries with standardised methodologies to refine optimal thresholds, formally establish its prognostic value, and further define its role in clinical decision-making.

## Supplementary Information

Below is the link to the electronic supplementary material.

Supplementary Material 1

## Data Availability

The data supporting the findings of this study are available from the corresponding author upon reasonable request.

## Abbreviations

ABI: Ankle-brachial Index
ACC_max_: Maximal Systolic Acceleration
AFR: Amputation-Free Survival
AMSTAR-2: A Measurement Tool to Assess Systematic Reviews version 2
AT_max_: Maximal Acceleration Time
AUC: Area Under the Curve
BKA: Below-the-Knee Amputation
CAD: Coronary Artery Disease
CKD: Chronic Kidney Disease
CLTI: Chronic Limb-Threatening Ischaemia
DBP: Diastolic Blood Pressure
DM: Diabetes Mellitus
DOR: Diagnostic Odds Ratio
DUS: Doppler Ultrasound
EDV: End Diastolic Velocity
ESRD: End Stage Renal Disease
GFR: Glomerular Filtration Rate
GRADE: Grading of Recommendations, Assessment, Development and Evaluation
HR: Heart Rate
HW: Healed Wound
I^2^: I Square Statistic
MI: Myocardial Infarction
NLBI: National Heart, Lung, and Blood Institute
NHW: Non-Healed Wound
NLR: Negative Likelihood Ratio
PAD: Peripheral Artery Disease
PAT: Pedal Acceleration Time
PI: Pulsatility Index
PLR: Positive Likelihood Ratio
PP: Pulse Pressure
PRISMA-DTA: Preferred Reporting Items for Systematic reviews and Meta-Analyses of Diagnostic Test Accuracy
PSV: Peak Systolic Velocity
PWV: Pulse Wave Velocity
RI: Resistivity Index
ROC: Receiver Operative Characteristic
SBP: Systolic Blood Pressure
SROC: Summary ROC
TASC: TransAtlantic Inter-Society Consensus
TBI: Toe Brachial Index
TP: Toe Pressure
TcPO2: Transcutaneous Oxygen Pressure
WIfI: Wound, Ischaemia, and foot Infection Classification
