# Supplementary material for: Pedal acceleration time as a diagnostic and prognostic tool in peripheral artery disease: systematic review and meta-analysis
Source: BMC Med Imaging. 2026 Jul 9;26:502. doi: 10.1186/s12880-026-02493-y (PMC13644277; doi:10.1186/s12880-026-02493-y)
Supplement: Supplementary file 1 [file 12880_2026_2493_MOESM1_ESM.docx]

ADDITIONAL FILE 1

| **Supplemental Table 1.1** | | | |
| --- | --- | --- | --- |
| **Section/topic** | **#** | **PRISMA-DTA Checklist Item** | **Reported on page #** |
| **TITLE / ABSTRACT** | | |  |
| Title | 1 | Identify the report as a systematic review (+/- meta-analysis) of diagnostic test accuracy (DTA) studies. | Page 1, Title: “Pedal Acceleration Time as a Diagnostic and Prognostic Tool in Peripheral Artery Disease: Systematic Review and Meta-Analysis” |
| Abstract | 2 | Abstract: See PRISMA-DTA for abstracts. |  |
| **INTRODUCTION** | | |  |
| Rationale | 3 | Describe the rationale for the review in the context of what is already known. | Page 6, Paragraph 2: “The ankle-brachial index (ABI) and Toe-Brachial Index is a well-known, non-invasive hemodynamic ratio, and is recommended as the first-line diagnostic test for PAD in the majority of societal guidelines (4, 6). However, ABI measurements might be unreliable in the presence of medial artery calcification, leading to falsely elevated (>1.40) ABI, and diagnostic uncertainty, particularly in patients with diabetes mellitus, chronic kidney disease, or advanced arterial stiffness (1, 6, 7). This limitation encourages clinicians to introduce complementary perfusion metrics into the evaluation of patients with lower-limb ischemia.” |
| Clinical role of index test | D1 | State the scientific and clinical background, including the intended use and clinical role of the index test, and if applicable, the rationale for minimally acceptable test accuracy (or minimum difference in accuracy for comparative design). | Page 6-7, Paragraph 3: “PAT is a Doppler ultrasound-derived parameter that measures, in milliseconds, the interval from the onset of systolic flow to the systolic peak in distal pedal arteries, thereby quantifying the hemodynamic delay caused by proximal arterial stenosis or occlusion (8, 9) (…) Previous studies have shown that PAT may allow grading of limb ischemia severity and may be associated with clinically relevant outcomes, such as wound healing and limb salvage, even in cases of medial calcification or non-compressive vessels (8, 13). Nevertheless, existing evidence is largely derived from small, retrospective, single-centre studies, and has not been comprehensively evaluated” |
| Objectives | 4 | Provide an explicit statement of question(s) being addressed in terms of participants, index test(s), and target condition(s). | Page 7, Paragraph 4: “Therefore, this systematic review and meta-analysis of patients with lower-limb ischemia aims to evaluate PAT for assessment of ischemia, in comparison with established perfusion tools such as ABI, thereby assessing diagnostic and prognostic performance. Secondary outcomes include assessing the prognostic value of PAT in predicting wound healing outcomes, evaluating its prognostic role in stratifying amputation risk, and to determine its utility as a monitoring tool for revascularization adequacy. Through these objectives, the present study seeks to clarify the potential role of PAT as a complementary tool for lower-limb perfusion assessment.” |
| **METHODS** | | |  |
| Protocol and registration | 5 | Indicate if a review protocol exists, if and where it can be accessed (e.g., Web address), and, if available, provide registration information including registration number. | Page 7; Paragraph 1: “The review protocol has been registered at Prospero (reference: CRD420251035946).” |
| Eligibility criteria | 6 | Specify study characteristics (participants, setting, index test(s), reference standard(s), target condition(s), and study design) and report characteristics (e.g., years considered, language, publication status) used as criteria for eligibility, giving rationale. | Section *Selection Criteria*, Page 8; Paragraphs 1-3: “Inclusion criteria comprised original human studies (except for systematic reviews and case series under 10 patients), evaluating PAT in the context of PAD. Eligible studies assessed PAT either as a diagnostic tool for lower-limb ischemia or as a prognostic marker for limb-related outcomes, irrespective of whether PAT was measured pre-operatively, post-operatively, or at a single time point. Studies reporting corresponding ABI values were included to allow correlation with PAT-derived perfusion metrics. Studies providing both pre- and post-operative PAT measurements were identified to explore associations between changes in PAT and clinical outcomes including wound healing, limb salvage, major amputation, and revascularization response. No restrictions were applied regarding publication date or language. Clinically significant lower-limb ischemia was defined according to the reference standard applied in each primary study. For the diagnostic meta-analysis, ABI-defined ischemia, using study-specific thresholds consistent with guideline-recommended cut-offs, was treated as the reference standard, recognizing that ABI is an imperfect surrogate, particularly in patients with non-compressible vessels.” |
| Information sources | 7 | Describe all information sources (e.g., databases with dates of coverage, contact with study authors to identify additional studies) in the search and date last searched. | Section *Search Strategy*, Page 8; Paragraphs 1-2: “A systematic search was performed in three databases: Medline via PubMed search, Scopus, and Web of Science, in August 2025. The full search strategy, including search queries and keywords is provided in Additional File 1 (Supplemental Table 1). Additionally, the reference lists of the included primary studies and relevant available systematic reviews were screened (snowballing) for any further articles of possible interest.” |
| Search | 8 | Present full search strategies for all electronic databases and other sources searched, including any limits used, such that they could be repeated. | Additional File 1, Supplemental Table 1 |
| Study selection | 9 | State the process for selecting studies (i.e., screening, eligibility, included in systematic review, and, if applicable, included in the meta-analysis). | Section *Study selection and data extraction*, Page 9, Paragraphs 1-2: “After duplicates removal, two authors (MRF and ES) have independently participated in the study selection. Disagreements on the choices were solved by the intervention of a third author (JRN). First, studies were selected by title and abstract, followed by full-text assessment. Studies were excluded if they involved patients without PAD or patients exclusively suffering from acute limb ischaemia, or if PAT was not assessed in relation to ABI, wound healing, or major amputation. Case reports, editorials, narrative reviews, grey literature, and conference abstracts were excluded. When full texts were not publicly available, authors were contacted. MRF and ES also performed a full-text assessment. The selected studies were carefully revised to avoid repeated populations. In the case of duplicate populations, the most detailed analysis was included.” |
| Data collection process | 10 | Describe method of data extraction from reports (e.g., piloted forms, independently, in duplicate) and any processes for obtaining and confirming data from investigators. | Section *Study selection and data extraction*, Page 9, Paragraph 3: “Data extraction was independently conducted by two authors (MRF and ES) using a purposely built form.” |
| Definitions for data extraction | 11 | Provide definitions used in data extraction and classifications of target condition(s), index test(s), reference standard(s) and other characteristics (e.g. study design, clinical setting). | Section *Study selection and data extraction*, Pages 9-10, Paragraphs 3-4: “Extracted study characteristics included year of publication, country, recruitment centre, study design, recruitment period, number of participants and limbs undergoing PAT measures, and follow up duration. Participant demographics included age, gender distribution, and prevalence of cardiovascular comorbidities. These data are available at Table 1 and Table 2. PAT methodology data included analysed arteries, PAT protocols with ABI correlated categories, comparative perfusion assessments including ABI and transcutaneous oxygen pressure (TcPO2), clinical staging systems, such as WIfI and Rutherford (when available) and pre- and post-operative PAT (when available). Extracted outcomes included wound healing rates at short-term (30 days) and long-term wound closure (3-12 months) follow-up, major amputation incidence and corresponding PAT value and wound healing threshold PAT values. Data from diagnostic accuracy of PAT compared to ABI were extracted to enable meta-analysis of the primary endpoint. The completed data extraction sheet is provided on Table 3 and Table 4. Given the heterogeneity in study populations and analytical methodologies, information on covariates considered for confounding adjustment in the original studies is reported on Supplemental Table 2, while study-specific inclusion and exclusion criteria are detailed on Supplemental Table 3. Both are available in the Additional File 1.” |
| Risk of bias and applicability | 12 | Describe methods used for assessing risk of bias in individual studies and concerns regarding the applicability to the review question. | Section *Assessment of study quality, Page 10, Paragraph 1: “*Concerning qualitative assessment, the National Heart, Lung, and Blood Institute (NHLBI) Study Quality Assessment Tool for observational cohort and cross-sectional studies (2021) was used (16) (…)The quality of evidence for the included articles was evaluated using the Grading of Recommendations, Assessment, Development, and Evaluation (GRADE) approach (17). Articles were classified into four levels of quality (high, moderate, low, and very low)”; |
| Diagnostic accuracy measures | 13 | State the principal diagnostic accuracy measure(s) reported (e.g. sensitivity, specificity) and state the unit of assessment (e.g. per-patient, per-lesion). | Section *Quantitative synthesis*, Page 11, Paragraph 2: “diagnostics random-effects model was preferred to obtain summary sensitivity, specificity, positive likelihood ratio (PLR), negative likelihood ratio (NLR), and diagnostic odds ratio (DOR)”; Unit of assessment: Per limb |
| Synthesis of results | 14 | Describe methods of handling data, combining results of studies and describing variability between studies. This could include, but is not limited to: a) handling of multiple definitions of target condition. b) handling of multiple thresholds of test positivity, c) handling multiple index test readers, d) handling of indeterminate test results, e) grouping and comparing tests, f) handling of different reference standards | Section *Quantitative Synthesis*, Page 10, Paragraph 1: “A diagnostic test accuracy meta-analysis using a bivariate random-effects meta-analyses approach was performed (…) ABI was considered the reference standard against which PAT (index test) was compared (…) Heterogeneity was evaluated through the I^2^ Q statistic-related measure of variance, and a diagnostics random-effects model was preferred to obtain summary sensitivity, specificity, positive likelihood ratio (PLR), negative likelihood ratio (NLR), and diagnostic odds ratio (DOR). Forest plots were used to display results from the meta-analysis. Sources of heterogeneity were assessed by leave-one-out sensitivity meta-analysis. Assessed covariates included the publication year, participants’ mean age, percentage of male participants, percentage of patients with arterial hypertension, dyslipidaemia, diabetes, or coronary artery disease, percentage of chronic kidney disease and use of antiplatelet therapy. Publication bias was assessed using funnel plots. Multivariable meta-regression analysis was not possible to perform because of an insufficient number of primary studies (<10)” Data handling definitions: described in Study selection and data extraction (Page 9); Synthesis/combination and variability: Quantitative Synthesis (Page 10). |

| **Supplemental Table 1.1 (cont.)** | | | |
| --- | --- | --- | --- |
| **Section/topic** | **#** | **PRISMA-DTA Checklist Item** | **Reported on page #** |
| Meta-analysis | D2 | Report the statistical methods used for meta-analyses, if performed. | Section *Quantitative Synthesis*, Pages 10-11, Paragraph 2-3: “A diagnostic test accuracy meta-analysis using a bivariate random-effects meta-analyses approach was performed (…) a diagnostics random-effects model was preferred to obtain summary sensitivity, specificity, positive likelihood ratio (PLR), negative likelihood ratio (NLR), and diagnostic odds ratio (DOR)” |
| Additional analyses | 16 | Describe methods of additional analyses (e.g., sensitivity or subgroup analyses, meta-regression), if done, indicating which were pre-specified. | Section *Quantitative Synthesis*, Page 11, Paragraph 3: “a leave-one-out sensitivity meta-analysis was conducted to evaluate the robustness of the pooled estimates and assess the influence of individual studies on the overall results, excluding each study in turn.” |
| **RESULTS** | | |  |
| Study selection | 17 | Provide numbers of studies screened, assessed for eligibility, included in the review (and included in meta-analysis, if applicable) with reasons for exclusions at each stage, ideally with a flow diagram. | Section *Search Results, Page 12, Paragraph 1*: “After the database search and duplicate exclusion, a total of 81 studies were screened. Upon title and abstract screening, 50 studies were excluded. For full-text assessment, 31 studies were eligible; 13 were excluded, and 1 was not retrieved (Figure 1). The criteria for exclusion upon full-text assessment were repeated patient population (N=2), intervention not aligned with review scope (N=9), insufficient number of participants (N=1) and study design not eligible (N=1). Thus, a total of 17 published articles were included in this systematic review. No studies were excluded from the systematic review or meta-analysis solely on the basis of their quality assessment score.”; Section *Main Findings and Meta-analysis*, Page 13, paragraphs 3-4: “Of the 17 studies included in the systematic review, only 4 provided data suitable for quantitative diagnostic meta-analysis. The remaining 13 studies were excluded from pooled analysis because they did not report paired sensitivity and specificity estimates against a standardised reference standard, did not use ABI as a formal comparator in a format permitting 2×2 contingency table construction, reported PAT exclusively as a continuous or categorical prognostic variable, or assessed acceleration parameters at proximal rather than pedal arterial levels. This selection criterion was applied to ensure methodological homogeneity of the pooled diagnostic estimates. In total, 4 primary studies reported extractable diagnostic data, suitable for quantitative synthesis (9-11, 13). One study (13) reported separate estimates for diabetic and non-diabetic subgroups, yielding five analytical datasets.” |
| Study characteristics | 18 | For each included study provide citations and present key characteristics including a) participant characteristics (presentation, prior testing), b) clinical setting, c) study design, d) target condition definition, e) index test, f) reference standard, g) sample size, h) funding sources | Section *Description of studies*, Page 12, Paragraph 1: “Among the 17 studies included in this systematic review, 8 were cross-sectional (10, 13, 22-27), 1 was a prospective observational study (28), and other 3 were retrospective observational (8, 11, 12). There were also 4 cohorts (29-32), 3 of them being prospective (29, 31, 32), 1 retrospective (30) and 1 case-control study (33). The included publications reported studies performed in 14 different countries within 5 continents – 5 from North America (8, 24, 26, 31, 32), 3 from South America (10, 13, 23), 6 from European (11, 12, 22, 25, 28, 30), 2 studies from Asia (27, 33) and 1 from Oceania (29). One study was conducted across both North America and Oceania (26). A total of 1749 patients were assessed, with a minimum of 28 and a maximum of 265 per study, and the mean participants’ age was 70.4 years. The percentage of male participants was 65,3% (n=1142). Demographics and comorbidities of the populations included in the studies are summarised in Table 2. ” |
| Risk of bias and applicability | 19 | Present evaluation of risk of bias and concerns regarding applicability for each study. | Section *Studies Quality*, Page 15, Paragraph 1: “The risk of bias in the selected articles is displayed in Figure 4a. The overall judgement, per evaluated item, regarding observational cohorts is shown in Figure 4b (…) the overall methodological quality was moderate, with substantial variability in specific domains.” |
| Results of individual studies | 20 | For each analysis in each study (e.g. unique combination of index test, reference standard, and positivity threshold) report 2x2 data (TP, FP, FN, TN) with estimates of diagnostic accuracy and confidence intervals, ideally with a forest or receiver operator characteristic (ROC) plot. | Section *Main Findings and Meta-analysis*, Page 13-14, Paragraphs 2-4: Study-specific estimates of sensitivity, specificity, positive and negative likelihood ratios, and diagnostic odds ratio are presented in Figures 2a, 2b, and 2c |
| Synthesis of results | 21 | Describe test accuracy, including variability; if meta-analysis was done, include results and confidence intervals. | Section *Main Findings and Meta-analysis*, Page 13, Paragraphs 3-4: “Across these datasets, pooled sensitivity of PAT for detecting clinically significant lower-limb ischemia, according to ABI-based definitions, was 0.72 (95% CI 0.68–0.76), with individual study estimates ranging from 0.66 to 0.83 (Figure 2a). Specificity was consistently high across cohorts, yielding a pooled value of 0.84 (95% CI 0.79–0.88). Between-dataset heterogeneity for both parameters was minimal (Figure 2a). The pooled PLR was 4.38 (95% CI 3.35–5.73), indicating that higher PAT values were associated with a meaningful increased in the post-test probability of significant ischemia. Correspondingly, the NLR was 0.25 (95% CI 0.19–0.33), demonstrating that lower PAT values were associated with a substantial reduction in the likelihood of clinically significant limb ischaemia. Both PLR and NLR showed low heterogeneity (I² = 0%), supporting consistency across diverse patient groups, including diabetic and non-diabetic subpopulations (Figure 2b). The I² value of 0% for positive and negative likelihood ratios indicates negligible between-study heterogeneity, consistent with the physiological stability of the PAT measurement across diverse patient populations and acquisition settings. The combined DOR was 14.85 (95% CI 10.02–22.01), reflecting strong overall diagnostic discriminatory performance. Individual study DORs varied substantially, ranging from 5.01 to 50.56, which is expected given the differences between population’s profiles and prevalence of advanced PAD. However, the pooled estimate remained stable under random-effects modelling (Figure 2c)” |
| Additional analysis | 23 | Give results of additional analyses, if done (e.g., sensitivity or subgroup analyses, meta-regression; analysis of index test: failure rates, proportion of inconclusive results, adverse events). | Section *Quantitative Synthesis*, Page 11, Paragraph 3: “Multivariable meta-regression analysis was not possible to perform because of an insufficient number of primary studies (<10).” |
| **DISCUSSION** | | |  |
| Summary of evidence | 24 | Summarize the main findings including the strength of evidence. | Page 16, Paragraph 1: “PAT demonstrated consistent diagnostic performance and robust discriminatory ability for detecting clinically significant lower-limb ischemia. PAT showed high specificity, robust likelihood ratios, and a diagnostic odds ratio close to 15, supporting reliable identification of ischemia, including in clinical contexts where ABI measurements may appear normal or be unreliable. The pooled diagnostic indices yielded a sensitivity of 0.72 (95% CI 0.68-0.76), specificity of 0.84 (95% CI 0.79-0.88), a PLR of 4.38 (95% CI 3.35-5.73), an NLR of 0.25 (95% CI 0.19-0.33), and a DOR of 14.85 (95% CI 10.02-22.01), supporting a strong association between PAT measurements, ABI-defined ischemia, and clinically relevant lower-limb perfusion deficits.” |
| Limitations | 25 | Discuss limitations from included studies (e.g. risk of bias and concerns regarding applicability) and from the review process (e.g. incomplete retrieval of identified research). | Pages 20-21, Paragraph 9: “This systematic review and meta-analysis, while providing comprehensive evidence on the diagnostic and prognostic value of PAT for lower-limb ischaemia, has several methodological constraints that must be considered. First, the number of eligible studies was limited, and sample sizes varied considerably, reducing statistical power and precluding subgroup or meta-regression analysis focused on specific arteries, acquisition settings, or reference standards. Nevertheless, the relatively narrow confidence intervals of pooled sensitivity, specificity, likelihood ratios, and diagnostic odds ratio, together with low to moderate heterogeneity, support the internal consistency of the summary estimates. Second, variability was observed across studies in the clinical application of PAT, including the arterial segments evaluated, the thresholds used to define abnormal values, and the reference standards applied for ischaemia classification. Although this variability may limit direct comparability and introduce spectrum or verification bias, most included studies enrolled patients presenting with clinical features such as non-healing ulcers or tissue loss, consistent with advanced ischaemia and CLTI. Importantly, higher PAT values were consistently associated with greater disease severity and poorer wound-related outcomes, suggesting a robust underlying physiological signal across diverse patient profiles, including those in whom ABI is less reliable. The use of ABI as the reference standard, while pragmatic and guideline-endorsed, introduces a risk of differential misclassification bias. In patients with incompressible vessels, ABI may underestimate ischaemia severity, leading to false-negative reference classifications that would artificially reduce PAT's apparent sensitivity and potentially overestimate its specificity. This suggests that the pooled diagnostic accuracy estimates reported here may be conservative, and that PAT's true discriminative performance, specifically in diabetic and calcified vessel populations, might exceed what the current meta-analysis captures. Furthermore, of the 17 studies included in the systematic review, only 4 provided data in a format suitable for quantitative diagnostic meta-analysis, as the remaining studies did not report paired sensitivity and specificity estimates against a standardized reference, precluding their inclusion in the pooled analysis. This discrepancy is a recognized methodological limitation of diagnostic test accuracy reviews and further underscores the need for standardized outcome reporting in future PAT studies. Evidence on prognostic performance for wound healing and limb outcomes was heterogeneous and could not be pooled. Finally, incomplete reporting of methodological aspects, such as blinding procedures and whether PAT thresholds were prespecified or derived from ROC analysis, may have introduced observer or selection bias. Other significant limitation of this meta-analysis is the small number of studies eligible for quantitative diagnostic synthesis. This substantially limits the generalizability of the pooled estimates, precludes meaningful subgroup analyses and meta-regression, and rendered formal funnel plot assessment of publication bias unfeasible. Few other short- and long-term outcomes were assessed, which deprived the study of PAT association, such as wound healing.” |
| Conclusions | 26 | Provide a general interpretation of the results in the context of other evidence. Discuss implications for future research and clinical practice (e.g. the intended use and clinical role of the index test). | Pages 21-22, Paragraph 10: “Future research should prioritize large, prospective, multicentre analysis of standardized PAT protocols, pre-specified thresholds, and clearly defined limb-based outcomes, directly comparing PAT with ABI, TcPO2, and other perfusion metrics. While ABI remains an established role in the initial evaluation of PAD, from the authors' perspective, PAT offers particular advantages over ABI in four clinical scenarios: patients with diabetes mellitus and medial arterial calcification, where falsely elevated ABI values render pressure-based assessment unreliable; patients with chronic kidney disease or end-stage renal disease on dialysis, who share a similar calcification burden; patients with established CLTI and tissue loss requiring serial perfusion monitoring to assess revascularisation adequacy and guide wound management; and patients with incidentally identified non-compressible vessels (ABI >1.40), in whom PAT should be considered a first-line alternative rather than a secondary investigation.” |
| **FUNDING** | | |  |
| Funding | 27 | For the systematic review, describe the sources of funding and other support and the role of the funders. | Section *Funding Sources*, Page 15, Paragraph 1: “The author(s) disclosed receipt of the following financial support for the research, authorship, and/or publication of this article: This article was supported by National Funds through FCT - Fundação para a Ciência e a Tecnologia,I.P., within CINTESIS, R&D Unit (reference UIDB/4255/2020) and within the scope of the project RISE, Associated Laboratory (reference LA/P/0053/2020) |

| **Supplemental Table 1.2** | | | |
| --- | --- | --- | --- |
| **Section/topic** | **#** | **PRISMA-DTA for Abstracts Checklist item** | **Reported on page #** |
| **TITLE and PURPOSE** | | |  |
| Title | 1 | Identify the report as a systematic review (+/- meta-analysis) of diagnostic test accuracy (DTA) studies. | Page 1: Pedal Acceleration Time as a Diagnostic and Prognostic Tool in Peripheral Artery Disease: Systematic Review and Meta-Analysis |
| Objectives | 2 | Indicate the research question, including components such as participants, index test, and target conditions. | Page 4, Paragraph 3: “to perform a systematic review and meta-analysis evaluating PAT’s diagnostic accuracy compared with ABI in detecting lower-limb ischaemia. Secondary objectives included assessing PAT’s prognostic value for wound healing and amputation risk.” |
| **METHODS** | | |  |
| Eligibility criteria | 3 | Include study characteristics used as criteria for eligibility. | Page 4, Paragraph 4: “studies evaluating PAT values and wound healing in PAD patients”; more information about eligibility criteria is detailed in main text. |
| Information sources | 4 | List the key databases searched and the search dates. | Page 4, Paragraph 4: “Three electronic databases MEDLINE, Scopus, and Web of Science, were systematically searched” |
| Risk of bias & applicability | 5 | Indicate the methods of assessing risk of bias and applicability. | Page 4, Paragraph 4: “Study quality was assessed using the National Heart, Lung, and Blood Institute (NHLBI) Study Quality Assessment Tool for observational cohort and cross-sectional designs.” |
| Synthesis of results | A1 | Indicate the methods for the data synthesis. | Page 4, Paragraph 4: “Pooled diagnostic indices, including sensitivity, specificity, positive and negative likelihood ratios, and diagnostic odds ratio with 95% confidence intervals were calculated.” |
| **RESULTS** | | |  |
| Included studies | 6 | Indicate the number and type of included studies and the participants and relevant characteristics of the studies (including the reference standard). | Page 5, Paragraph 5: “Seventeen studies (1749 patients) were included as following: 8 observational cross-sectional, 1 observational prospective, 3 observational retrospective, 4 cohort (3 retrospective, 1 prospective), and 1 case-control study.” |
| Synthesis of results | 7 | Include the results for the analysis of diagnostic accuracy, preferably indicating the number of studies and participants. Describe test accuracy including variability; if meta-analysis was done, include summary results and confidence intervals. | Page 5, Paragraph 5: “Seventeen studies (1749 patients) (…)PAT demonstrated good diagnostic accuracy with pooled 0.72 (95% CI 0.68-0.76) sensitivity, 0.84 (95% CI 0.79-0.88) specificity, 4.38 (95% CI 3.35-5.73) positive likelihood ratio, 0.25 (95% CI 0.19-0.33) negative likelihood ratio, and 14.85 (95% CI 10.02-22.01) diagnostic odds ratio, indicating robust discrimination compared to ABI.” |
| **DISCUSSION** | | |  |
| Strengths and limitations | 9 | Provide a brief summary of the strengths and limitations of the evidence | Reported in main text |
| Interpretation | 10 | Provide a general interpretation of the results and the important implications. | Page 5, Paragraph 6: “PAT accurately detected clinically significant lower-limb ischaemia compared to ABI. Faster PAT values correlated to higher ABI and favorable wound healing outcomes, whereas prolonged PAT indicated higher risk of non-healing wounds and major amputation. These results support PAT as a useful duplex-based complement to existing perfusion tests.” |
| **OTHER** | | |  |
| Funding | 11 | Indicate the primary source of funding for the review. | Reported in main text |
| Registration | 12 | Provide the registration number and the registry name | Reported in main text |

| **Supplemental Table 2** | | |
| --- | --- | --- |
| **Bibliographic source** | **Search term** | **No of reports**  8/2025 |
| MEDLINE | (“Peripheral Arterial Disease” OR (intermittent claudication[Title/Abstract]) OR (lower extremity artery disease[Title/Abstract]) OR (peripheral artery disease[Title/Abstract]) OR (peripheral vascular disease[Title/Abstract]) OR (peripheral arterial disease[Title/Abstract]) OR (peripheral obliterative arteriopathy[Title/Abstract]) OR (arterial occlusive disease[Title/Abstract]))  AND  ((“Acceleration Time” OR "Peak Systolic Velocity" OR “Maximal Systolic Acceleration")   AND  (Pedal OR Plantar)) | 27 |
| Web of Science | ("Peripheral Arterial Disease" OR (intermittent AND claudication) OR (lower AND extremity AND artery AND disease) OR (peripheral AND artery AND disease) OR (peripheral AND vascular AND disease ) OR (peripheral AND arterial AND disease) OR (peripheral AND obliterative AND arteriopathy) OR (arterial AND occlusive AND disease))   AND   ("Acceleration Time" OR "Peak Systolic Velocity" OR “Maximal Systolic Acceleration")  AND  (pedal OR plantar) | 28 |
| SCOPUS | ("Peripheral Arterial Disease" OR (intermittent AND claudication) OR (lower AND extremity AND artery AND disease) OR (peripheral AND artery AND disease) OR (peripheral AND vascular AND disease ) OR (peripheral AND arterial AND disease) OR (peripheral AND obliterative AND arteriopathy) OR (arterial AND occlusive AND disease))   AND   ("Acceleration Time" OR "Peak Systolic Velocity" OR “Maximal Systolic Acceleration")  AND  (pedal OR plantar) | 37 |

| **Supplemental Table 3** | |
| --- | --- |
| **Author** | **Covariables used in adjusted models** |
| Arévalo-Zamora et al. | - |
| de Castro-Santos et al. | - |
| Del Valle et al. | - |
| Hart et al. | - |
| Junaidi et al. | - |
| Mendes-Pinto et al. | - |
| Ochoa-Ayón et al. | - |
| Pinelo et al. | - |
| Silva et al. | - |
| Sommerset et al. | - |
| Spiliopoulos et al. | - |
| Teso et al. | - |
| Trihan et al. | - |
| Trihan et al. | - |
| Tun et al. | - |
| Willems et al. | - |

| **Supplemental Table 4** | | |
| --- | --- | --- |
| **Authors** | **Inclusion Criteria** | **Exclusion Criteria** |
| Arévalo-Zamora et al. | Patients >18 years, of both sexes, who underwent arterial plethysmography and voluntarily agreed to PAT testing | Revascularization or vascular intervention on target limb, aortoiliac arterial disease; refusal to participate in the study |
| de Castro-Santos et al. | Patients with and without diabetes >18 years with CLTI | Patients <18 years; acute limb ischemia; peripheral aneurysms or blue toe disease; lateral plantar artery occlusion/nonpulsatile flow; elevated/incompressible ankle systolic pressure |
| Del Valle et al. | Indications for scanning were claudication in 63% and foot ulcerations in 37% | Patients that had a patent distal bypass graft were excluded |
| Hart et al. | Patients with lower-limb ulceration or gangrene of any severity, irrespective of infection or wound status | Age <18years; no wound present; with purely venous or mixed arteriovenous wound etiologies; isolated aorto-iliac PAD |
| Junaidi et al. | All patients with PAD who underwent BKA | Patients who died in the treatment period, ascending infections, congenital vascular abnormalities, and with hypoalbuminemia (<3.5 g/dl) |
| Mendes-Pinto et al. | CLTI classified as resting pain ischemic pain at rest or ischemic trophic ulcers on the lower limbs | Patients < 18 years; Advanced heart failure (NYHA IV); pregnant women; patients with ABI >1.3 |
| Ochoa-Ayón et al. | Atherosclerotic infrainguinal CLTI, WIfI 0/1 infection, candidates for limb salvage undergoing angioplasty, with PAT determined before and after the procedure | Not specified |
| Pinelo et al. | Diabetic patients from multidisciplinary diabetic foot clinic previously submitted to lower limb angiography | Not specified |
| Silva et al. | Patients referred to our department with PAD and lower limb wounds | Limbs with unmeasurable PAT |
| Sommerset et al. | Diabetic and current smoker >50 years, signs/symptoms of PAD (exertional leg pain, non-palpable pulses, lower limb dysaesthesis, colour changes, non-healing wounds, abnormal vascular screening, known/suspected vascular or aneurysm) | Vasospastic or connective tissue disorders (scleroderma or Raynaud’s disease); bilateral mastectomy or presence of arteriovenous fistula preventing any brachial blood pressure measurement; known allergy to ultrasound gel |
| Spiliopoulos et al. | CLTI patients with wounds, ischemia, and foot infection wound WIfI class ≥1 undergoing endovascular interventions | Concomitant iliac inflow or common femoral artery disease; preexisting or prescheduled major knee-level amputation; failing surgical bypass, extensive wounds |
| Teso et al. | Infra-inguinal disease who underwent arterial intervention; non-compressible ABI; who has had a PAT performed pre- and post-procedure | Pending amputation due to severe infection (WIfI infection class 2 and 3) |
| Trihan et al. | All patients who underwent DUS with measurements of AT_max_ and TBI | Age <18 years; protected adults; impossibility of either TP or AT_max_ measurements; occlusion of both DPA and LPA on the same lower limb. |
| Trihan et al. | Occlusive PAD | Age <18 years, toe or proximal amputation; protected adults; arterial revascularization on the included lower limb causing permeability from the aorta to the popliteal artery. |
| Tun et al. | All patients with PAT measurements (when there was inadequate or questionable assessment of foot perfusion) were included. | Not Specified |
| Willems et al. | Patients aged ≥18 years with a new-onset foot ulcer (i.e. Fontaine IV and neuropathic ulcers) | Patients on prednisone or chemotherapy; primary amputation due to severe tissue loss or advanced infection. |
| Yagyu et al. | Patients with angiography and DUS data of lower extremity arteries | Significant stenotic or occlusive lesions or history of a surgical procedure at the CFA or PopA |

AT_max_ : Maximal Acceleration Time; BKA: Below the Knee Amputation; CLTI: Chronic Limb Threatening Ischemia; CLI: Critical Limb Ischemia CFA: Common Femoral Artery; DUS: Doppler Ultrasound PAD; NYHA: New York Heart Association; PAD: Peripheral Artery Disease; PAT: Pedal Acceleration Time; PopA: Popliteal Artery; TBI: Toe Brachial Index; WIfI: Wound Ischemia Foot Infection classification.
